# Sirtuin 6 is a histone delactylase

**DOI:** 10.1016/j.jbc.2025.110795

**Published:** 2025-10-06

**Authors:** Garrison A. Nickel, Nicholas J. Pederson, Faheem, Zhenyu Yang, Jack Bulf, Katharine L. Diehl

**Affiliations:** 1 Department of Medicinal Chemistry, University of Utah, Salt Lake City, Utah, USA; 2 Juan Diego Catholic High School, Draper, Utah, USA

**Keywords:** histone deacetylase, sirtuin, histone modifications, lactic acid, epigenetics, histone lactylation

## Abstract

Histone lactylation (Kla) is a post-translational modification (PTM) that is derived from metabolic lactate. Histone Kla has been extensively studied in the field of inflammation resolution and macrophage polarization but has also been implicated in diverse cellular processes including differentiation, various wound repair phenotypes, and oncogenesis in several cancer models. While mechanistic connections between histone Kla and transcriptional changes have been studied in very limited contexts, general mechanistic details describing how regulation of gene expression by histone Kla occurs are scarce. It is hypothesized that histone Kla may be installed either through nonenzymatic means or by acetyltransferases like p300, and it is known that Class I HDACs and Sirtuins 1 to 3 can remove histone Kla. Here, we identified histone delactylase activity of the deacylase enzyme Sirtuin 6 (Sirt6), a member of the Class III HDAC family known to have roles in regulating metabolic homeostasis. We characterized the ability of Sirt6 to delactylate histones *in vitro* and in a mammalian cell culture model. We identified H3K9 and H3K18, canonical histone sites of Sirt6-catalyzed deacetylase activity, as sites of its delactylase activity. We also demonstrated that Sirt6 and the Class I HDACs exhibit some degree of non-overlapping delactylase activity, suggesting that they represent different cellular axes of regulating gene expression *via* controlling levels of histone Kla.

Histones, the primary protein component of eukaryotic chromatin organization, are heavily modified with a wide variety of post-translational modifications (PTMs) (1, 2). While PTMs such as histone lysine methylation (Kme) and lysine acetylation (Kac) have been extensively studied and their roles are relatively well-defined (3, 4, 5, 6), in recent years, mass spectrometric approaches have identified a host of novel histone PTMs which have not yet been fully characterized. These newly discovered PTMs, such as lysine β-hydroxybutyrylation (7, 8), succinylation (9, 10, 11), malonylation (10), and propionylation (12, 13), incorporate diverse primary metabolites into histone modifications. One prevalent hypothesis posits that these metabolite-derived PTMs are indicative of a regulatory axis through which high levels of metabolites can be ‘sensed’ by the cell and converted into a transcriptional response (14, 15, 16).

Originally discovered in 2019 (17), histone lysine lactylation (Kla) is a leading example of how cellular primary metabolite pools can influence gene expression by being directly incorporated into histone PTMs. Measurable increases in histone Kla result from treatment of cells with supplemental lactate or under conditions that bias metabolism towards glycolysis and lactate production (17, 18). Isotopic labelling experiments demonstrated that extracellular lactate and extracellular glucose are both incorporated into Kla modifications, directly linking cellular metabolism to histone modifications (17). There is some debate about the immediate source of histone lactylation, with some studies pointing to glyoxalate metabolism as a source for reactive lactyl moieties (19, 20). However, a recent study found that L-lactylation is the dominant stereoisomer of histone Kla present in cells, implicating lactyl-CoA as the precursor to histone lactylation (21). The concentration of lactyl-CoA detected in HepG2 cells under typical culture conditions (*i.e.*, no lactate supplementation, high glucose) is about 1000 times lower than that of acetyl-CoA, but recent studies have found that lactyl-CoA levels are highly sensitive to glucose metabolism in these same cells (21, 22). A body of recent evidence implicates histone Kla in a variety of biological processes and diseases, including oncogenesis and cancer progression (23, 24, 25, 26, 27, 28), differentiation (29), anti-inflammatory response (17, 18, 30, 31, 32), tissue repair (33), angiogenesis (34, 35), and metabolic diseases such as diabetes mellitus (36, 37). While there is evidence that histone Kla and Kac exhibit different genomic localization (17, 33), it remains unclear mechanistically how this occurs and to what extent Kla is functionally distinct from Kac. Additionally, since conditions typically used to stimulate or abrogate histone Kla do so by modulating glycolysis or lactate levels, it is difficult to deconvolute the effects of histone Kla from other signaling effects caused by extracellular and/or intracellular lactate (38, 39).

Histone lactylation can be installed by many of the same acetyltransferases which install histone acetylation, including p300, GCN5, and HBO1 (17, 40, 41). Emerging evidence suggests that these acyltransferase enzymes can cooperate with lactyl CoA-producing enzymes in the nucleus to catalyze efficient installation of Kla (41, 42). This cooperative behavior is one potential mechanism by which gene-specific deposition of histone Kla could be achieved. Several mechanisms by which histone Kla removal occurs have been described. Moreno-Yruela *et al.* showed that the class I histone deacetylases (HDACs) are able to remove histone Kla, although they also deacylated all other PTM substrates tested, exhibiting no obvious substrate selectivity (43). The Sirtuin family of NAD^+^-dependent deacetylases (also referred to as the class III HDACs) have recently been demonstrated to remove a variety of noncanonical histone acyl PTMs, especially Sirtuin 2 and Sirtuin 3 (23, 25, 44). Jennings *et al.* investigated the ability of the Sirtuins to remove the Kla modification from a non-histone peptide substrate and identified Sirtuin 2 (Sirt2) as a delactylase (44). Further research by Zu *et al.* revealed that Sirt2 also delactylates histones and that Sirt2 knockdown was sufficient to cause increased cellular accumulation of histone Kla in a neuroblastoma model (25). More recently, Du *et al.* demonstrated that Sirtuins 1 and 3 are capable of catalyzing lysine delactylation in histone and non-histone contexts (45). None of these studies identified the nuclear-localized members of the Sirtuin family, Sirtuins 6 and 7 (Sirt6 and Sirt7), as delactylases, but those that tested Sirt6 and Sirt7 all used peptide or single-histone substrates in their screens of deacylase enzymes. Experimental evidence indicates that Sirt6 exhibits very low deacylase activity on peptide and histone substrates but much higher deacylase activity on whole nucleosome substrates and that this behavior is dependent on Sirt6-histone and Sirt6-DNA interactions (46, 47, 48, 49, 50, 51). Several recent structural studies further support those biochemical studies (52, 53, 54). These findings led us to hypothesize that Sirt6 and/or Sirt7 may exhibit previously overlooked delactylase activity when tested in a nucleosomal context. Herein, we performed biochemical and cell-based assays that show that Sirt6 is a histone delactylase.

## Results

### Sirt6 exhibits NAD^+^-dependent delactylase activity *in vitro*

**Figure 1:**
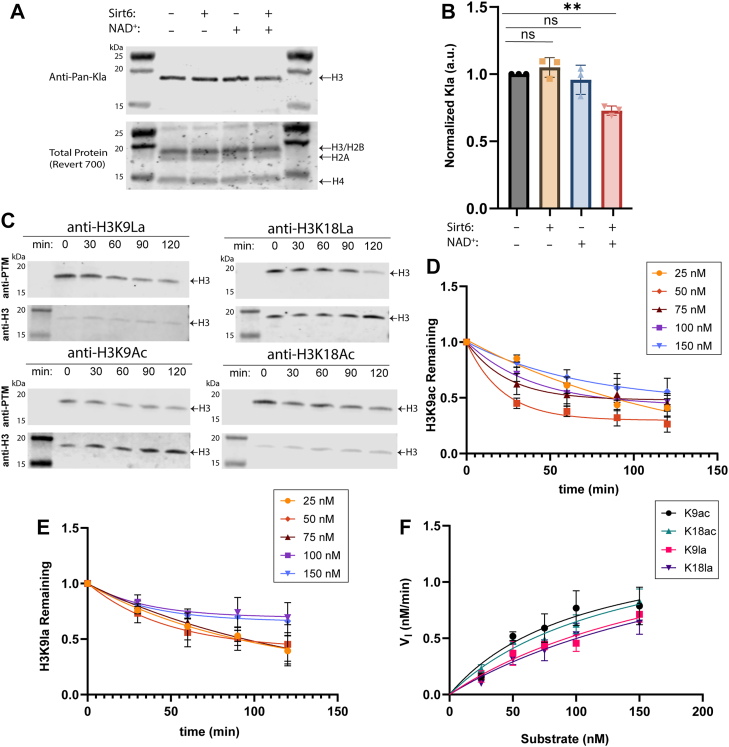
**Sirt6 exhibits histone delactylase activity *in vitro*.***A*, Western blot of histone Kla levels on nucleosomes isolated from HEK-293T cells that were incubated with recombinant Sirt6 and/or NAD^+^. *B*, quantitation of the blot from *panel A*. Densitometry data were corrected based on the total protein stain, then normalized to the “no NAD^+^/no Sirt6” control condition. Groups were compared using a one-way ANOVA followed by Tukey’s *post hoc* test. n = 3 technical replicates, error plotted as S.D. p (−/− v. +/+) = 0.006. *C*, Western blot analysis of time courses of enzymatic deacylation reactions using 50 nM substrate nucleosome, 100 nM Sirt6, and 1 mM NAD^+^. *D*, quantitation of Western blot data for the H3K9Ac substrate. Densitometry data were corrected based on the H3 loading control, then normalized to the t = 0 time point. *E*, quantitation of Western blot data for the H3K9La substrate. Data was processed as in *panel D*. *F*, Michaelis–Menten plot showing V_I_ values plotted as a function of substrate concentration for each PTM substrate. V_I_ was calculated for each substrate concentration by fitting the first three time points using standard linear regression. For *panels D*, *E*, and *F*, n = 3 technical replicates, error plotted as S.D.

We first tested whether Sirt6 is capable of delactylating histones *in vitro*. We isolated nucleosomes from HEK-293T cells that were treated with 25 mM sodium L-lactate (to stimulate Kla) *via* detergent-free lysis, sonication, and digestion with micrococcal nuclease (55). These nucleosomes were then incubated with recombinantly expressed Sirt6 (full-length, human sequence), NAD^+^, or both, and histone Kla levels were measured using a pan-α-Kla antibody (Fig. 1, *A* and *B*). We observed that histone Kla levels were decreased only in the presence of Sirt6 and NAD^+^, leading us to conclude that the observed delactylation was due to the NAD^+^-dependent deacylase activity of the recombinant Sirt6.

To assess which histone sites are delactylated by Sirt6, we incubated HEK-293T nucleosomes with recombinant Sirt6 and NAD^+^ and analyzed the samples using a panel of site-specific Kla and Kac antibodies: H3K9ac, H3K9la, H3K18ac, H3K18la, H4K12la, and H3K56la (Fig. S1) (21). We were unable to detect several PTMs with these antibodies using the HEK-293T nucleosomes, so we also isolated nucleosomes from Sirt6−/− U2OS cells (“S6KO”) using the same protocol as for the HEK-293T nucleosomes. We detected several of the “missing” PTMs in this cell line, leading us to conclude that those PTM levels were below the antibody’s limit of detection in the HEK-293T cells. Other PTMs that were detected using HEK-293T nucleosomes were below the antibody’s limit of detection on the U2OS nucleosomes. We found that Sirt6 deacetylates and delactylates at H3K9 (Fig. S1, *A* and *B*) and H3K18 (Fig. S1*B*) but did not detect delactylation of H3K56 or H4K12 (Fig. S1*B*) in the assay.

### Sirt6 exhibits similar deacetylase and delactylase activity *in vitro*

To quantify the ability of Sirt6 to delactylate histones relative to its canonical deacetylase activity, we performed *in vitro* kinetics assays similar to previously published protocols (52). We prepared a panel of singly-modified mononucleosomes with H3K9ac, H3K9la, H3K18ac, or H3K18la by semisynthesis. These nucleosomes were incubated with recombinant Sirt6 (human, full-length) and NAD^+^ across a 2-h time course at varying concentrations of nucleosome, and the extent of PTM removal was quantified using Western blot (Figs. 1, *C*–*E* and S2, *A*, *B*). The intensities from the kinetics data, corrected based on a loading control, were normalized as a fold change from the t = 0 condition, then multiplied by the starting concentration of substrate to calculate the amount of substrate remaining at each time point. Since the rate of substrate consumption was approximately linear during the first hour of data collection for all four substrates, the first three data points for each substrate were fit using standard linear regression to calculate an initial rate of deacylation (V_I_, Figs. 1*F* and S2*C*). These V_I_ values were then plotted as a function of substrate concentration and fit using the Michaelis-Menten model to determine an apparent K_M,app_ and k_cat,app_ for each substrate (Table S1). Based on these data, we concluded that Sirt6 exhibits delactylase behavior. The kinetic parameters we determined were within the SEM of each other for the acetyl and lactyl PTMs tested at each site, so we conclude that there is no significant difference between Sirt6’s delactylase and deacetylase activity at the K9 or K18 sites. Based on our data, we would postulate that, if there is a slight difference in the rate of deacetylase and delactylase activities, this is primarily due to poorer binding of Sirt6 to the Kla substrates. We also expressed human, full-length Sirt7 and performed immunoblot assays profiling Sirt7 deacylase activity. We observed that Sirt7 exhibits deacetylase but not delactylase behavior at H3K18 (Fig. S2, *D*–*F*). As such, we did not proceed with the kinetics analysis of Sirt7.

### Sirt6 deletion increases cellular accumulation of histone Kla

**Figure 2:**
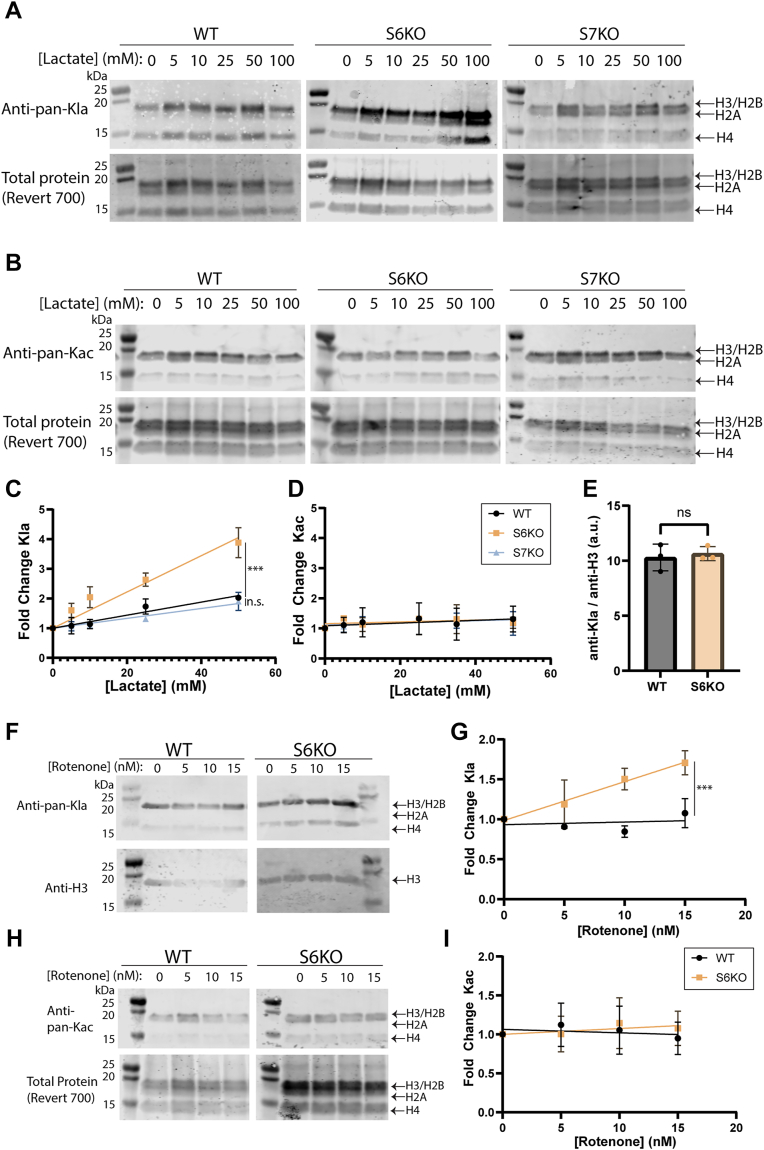
**Sirt6 depletion leads to histone Kla accumulation in cells.***A*, Western blot using a pan-lactyllysine antibody to analyze Kla levels on acid-extracted histones from wild type (“WT”), Sirt6 knockout (“S6KO”), and Sirt7 knockout (“S7KO”) U2OS cells in the presence of a titration of sodium L-lactate. Total protein was measured using a fluorescent total protein stain. *B*, as in *panel A* but using a pan-acetyllysine antibody to analyze levels of histone Kac. *C*, Western blots from *panel A* quantified by normalizing Kla signal to total protein then represented as a fold-change from the untreated condition. n = 3 biological replicates, error plotted as S.D. Slopes of linear regressions were compared using Welch’s *t* test. p (slope, S6KO v. WT) < 0.0001. p (slope, S7KO v. WT) = 0.39. *D*, Western blots from *panel B* quantified by normalizing Kac signal to total protein then represented as a fold-change from the untreated condition. For WT and S6KO, n = 3 biological replicates. For S7KO, n = 2 biological replicates. Error plotted as S.D. *E*, baseline histone lactylation in untreated cells was measured using a pan-Kla antibody as in *panel A* and normalized to the loading control. Note: these same data are plotted again in Figure 4*C*. Statistical analysis was performed using one-way ANOVA followed by Tukey’s *post hoc* test. Error plotted as S.D. *F*, Western blot using a pan-lactyllysine antibody (PTM BIO) to analyze Kla levels on acid-extracted histones from WT and S6KO U2OS cells in the presence of a titration of rotenone, a mitochondrial Complex I inhibitor. Total protein was measured using a fluorescent total protein stain (LI-COR). *G*, antibody signal was corrected based on the loading control signal, then normalized as a fold-change from the untreated condition. n = 3 biological replicates, error plotted as S.D. Slopes of linear regressions were compared using Welch’s *t* test, *p* = 0.0005. *H*, as in panel F but using the pan-acetyllysine antibody to measure levels of histone Kac. (*I*) Quantitation of data from *panel H*. Data were processed as in *panel G*. n = 3 biological replicates, error plotted as S.D.

To assess the histone delactylase activity of Sirt6 and Sirt7 in a cell culture model, we generated Sirt6(−/−) and Sirt7(−/−) U2OS cells (“S6KO” or “S7KO”) using CRISPR-Cas9. These cell lines were then treated with varying concentrations of sodium L-lactate for 24 h to stimulate histone Kla. Total Kla levels on acid-extracted histones from the cells were measured *via* immunoblotting and normalized to the [lactate] = 0 mM level within each cell line (Fig. 2, *A* and *B*). The S6KO cells exhibited a greater accumulation of histone Kla compared to the WT or S7KO cells upon lactate supplementation (Fig. 2*C*). We also measured levels of histone Kac on the extracted histones as a control, since supplementation with lactate should not cause an increase in histone acetylation (Fig. 2*D*) (17). As expected, none of the cell lines tested exhibited significant changes in bulk histone acetylation as a function of treatment with lactate. This result supports the selectivity of the pan-Kla antibody and is consistent with previously published data from other cell lines treated with sodium L-lactate (17). Even though the absence of Sirt6 led to higher accumulation of histone lactylation upon lactate supplementation compared to in the WT cells (Fig. 2*C*), the S6KO cells did not exhibit a difference in the baseline level of total histone Kla without lactate supplementation (Fig. 2*E*). Since we did not observe evidence of Sirt7-catalyzed delactylation in either our *in vitro* or cell-based assays, subsequent experiments focused solely on the activity of Sirt6.

Using the WT and S6KO U2OS cells, we performed an experiment in which Kla was stimulated using rotenone, a mitochondrial Complex I inhibitor, to increase intracellular lactate as previously described (17). Under these conditions, we again observed that S6KO cells accumulated histone Kla to a greater extent than WT cells (Fig. 2, *F* and *G*). In agreement with Zhang *et al.*, histone Kac levels were unaffected by rotenone treatment (Fig. 2, *H* and *I*) (17). We also repeated the lactate supplementation experiment with the addition of sodium oxamate (a lactate dehydrogenase LDH inhibitor). Under these conditions, the cells should not be able to produce lactate from metabolic pyruvate, and the supplemental lactate should not be able to be oxidized to pyruvate. Under these conditions, the S6KO cells again accumulated histone Kla to a higher extent than WT or S7KO cells (Fig. S3).

**Figure 3:**
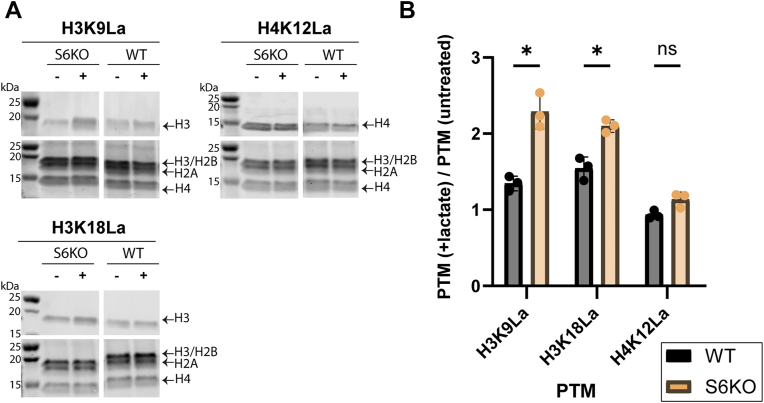
**Sites of Sirt6-catalyzed histone delactylation in cells.***A*, levels of various histone lactyl PTMs on acid-extracted histones from WT or S6KO U2OS cells were measured using site-specific antibodies (CST and PTM Bio). Total protein in each sample was measured using a fluorescent total protein stain (LI-COR). Samples from S6KO and WT cells were analyzed on the same membrane and are spliced here only to make direct comparisons more evident. The complete blots can be found in the Supporting Information. *B*, quantitation of data from *panel A*. Data were corrected based on the Revert 700 total protein stain, then presented as a fold-change from the untreated control condition. Statistical analysis was performed using Student’s 2-tailed *t* test and corrected for multiple hypothesis testing using the Holm-Šídák correction. n = 3 biological replicates, error plotted as S.D. H3K9La *p* = 0.013, H3K18La *p* = 0.019. Note: The same data in *panel B* is also plotted in Fig. S4.

To examine sites at which Sirt6 delactylates in cells, we measured acetylation and lactylation at various histone sites in the U2OS WT and S6KO cells using a panel of site-specific antibodies: H3K9la, H3K18la, H4K12la, and H3K56la (PTM BIO, Figs. 3 and S4). In the presence of exogenous lactate, the S6KO cells experienced a greater accumulation of H3K9la and H3K18la, but not of H4K12la, as compared to accumulation in the WT cells (Fig. 3). These data indicate that Sirt6 can remove Kla from H3K9 and H3K18 in cells, in line with our *in vitro* data (Fig. 1, *C*–*F*). We were unable to detect H3K56la in either cell line by immunoblot, even under lactate supplementation (data available as uncropped blots in Supporting Information).

### Sirt6 overexpression reduces cellular levels of histone Kla

**Figure 4:**
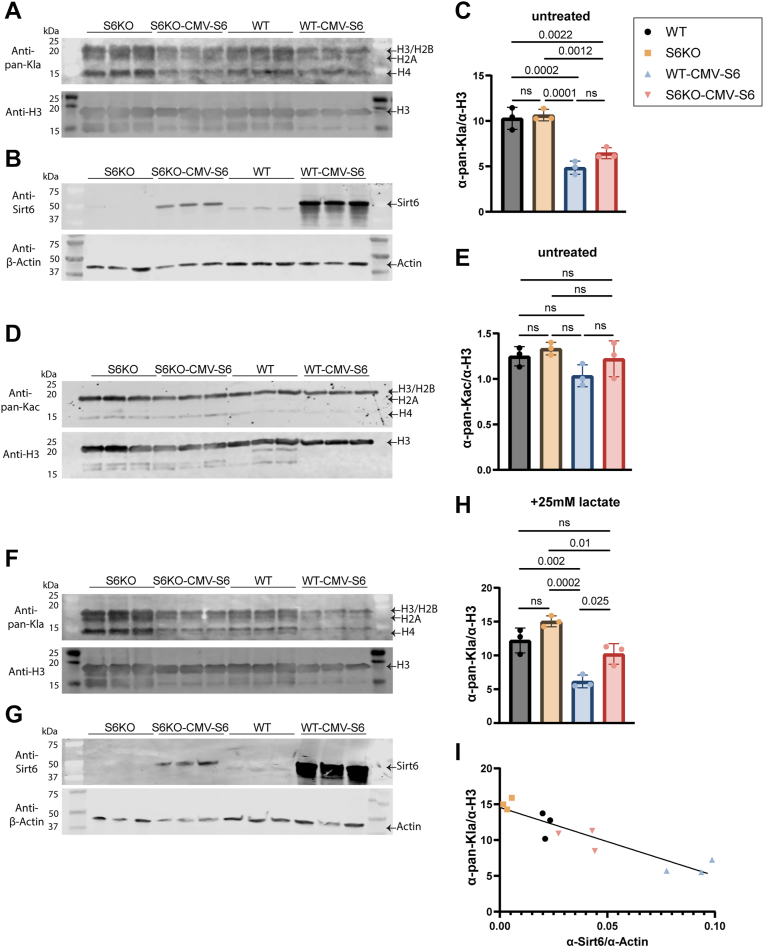
**Cellular histone Kla is reduced by overexpression of Sirt6.***A*, Western blot measuring histone Kla on acid-extracted histones in WT and S6KO U2OS cell lines not treated with supplemental L-lactate with and without overexpression of human Sirt6 under the control of a CMV promoter. WT histone H3 (CST) is used as a loading control. *B*, Western blot measuring Sirt6 in the soluble protein fraction from cell lines from *panel A*. β-Actin is used as a loading control. *C*, Kla signal from *panel A* was quantified and normalized to the loading control. Statistical analysis was performed using one-way ANOVA followed by Tukey’s *post hoc* test. n = 3 biological replicates, error plotted as S.D. *D*, histone Kac was measured using the same method and controls as in *panel A*. *E*, Kac data from *panel D* was quantified as in *panel C*. n = 3 biological replicates, error plotted as S.D. *F*, as in panel A, but the cells were treated with 25 mM sodium L-lactate for 24 h prior to histone extraction. *G*, As in *panel B*, but the cells were treated with 25 mM sodium L-lactate for 24 h prior to cell lysis. *H*, Kla signal from *panel F* was quantified and normalized to the loading control. *I*, inverse correlation of data from *panels F* and *G*. Sirt6 signal was normalized by dividing by the β-actin loading control. Kla signal was normalized as in *panel H*. The data were analyzed using a standard linear regression, R^2^ = 0.84. For all *panels*, *p*-values < 0.05 have been printed in the figure, *p*-values > 0.05 marked as ns.

We next sought to determine whether Sirt6 overexpression causes cells to exhibit decreased levels of histone Kla. We overexpressed 3xFLAG-tagged full-length human Sirt6 under the control of a CMV promoter in WT U2OS cells (“WT-CMV-S6,” = overexpression) and in the S6KO cells (“S6KO-CMV-S6,” = addback) (Fig. 4). Histones were extracted from untreated cells (*i.e.*, no lactate added) and from cells treated with 25 mM sodium L-lactate for 24 h, then analyzed for total histone Kla or Kac. Sirt6 levels in each cell line were measured to validate the overexpression and to quantify the amount of Sirt6. In both treated and untreated cells, histone Kla was decreased upon Sirt6 overexpression (WT-CMV-S6) compared to WT (and KO) cells (Fig. 4, *A*–*C*, *F*–*H*), supporting the hypothesis that Sirt6 can delactylate histones in the U2OS cells. In both treated and untreated conditions, we observed no baseline difference in global histone Kla (Fig. 4, *A*, *C*, *F* and *H*) or Kac (Fig. 4, *D* and *E*) in Sirt6 WT vs KO cells, Since there are multiple deacetylase and delactylase enzymes besides Sirt6 in human cells (25, 43, 45), we posit that these other enzymes compensate for the absence of Sirt6, which could significantly dampen the apparent effect of Sirt6, particularly when looking at the total histone Kac or Kla (rather than at specific sites) as we did in Figure 4. In cells treated with lactate, histone Kla was inversely proportional to the amount of Sirt6 expression (Fig. 4*I*), indicating Sirt6 histone delactylase activity.

### Sirt6 and class I HDACs exhibit additive histone delactylase activity

**Figure 5:**
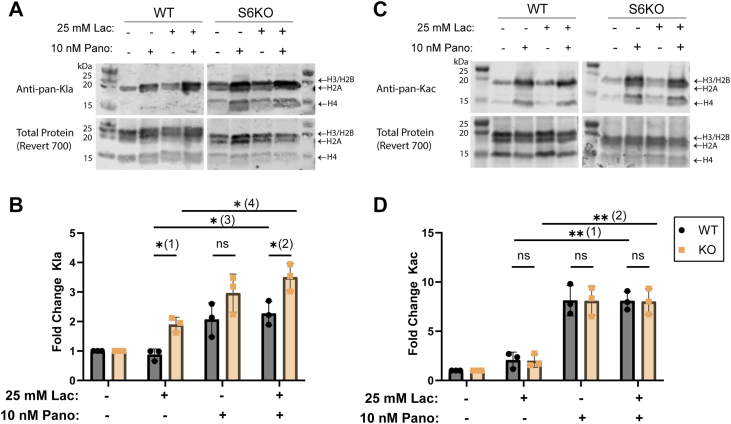
**Sirt6-and HDAC-catalyzed histone delactylation are additive.***A*, Western blot measuring histone Kla in WT and S6KO U2OS cells treated with sodium L-lactate and panobinostat, as indicated. A total protein stain (LI-COR) was used as a loading control. *B*, quantitation of data from *panel A*. Histone Kla signal was quantified using densitometry and corrected based on the total protein stain. Data are presented as a fold change from the untreated (-lactate, -panobinostat) condition. Statistical analysis was performed using Student’s 2-tailed *t* test and corrected for multiple comparisons using the Holm-Šídák correction. p_3_ and p_4_ were calculated using Student’s 2-tailed *t* test. *p*_1_ = 0.03, *p*_2_ = 0.02, *p*_3_ = 0.005, *p*_4_ = 0.002. n = 3 biological replicates, error plotted as S.D. *C*, histone Kac was measured using the same methods and controls as in *panel A*. *D*, quantitation of data from *panel C*. Data was processed as in *panel B*. *p*_1_ = 0.001, *p*_2_ = 0.002. n = 3 biological replicates, error plotted as S.D.

The Zn^2+^-dependent Class I HDACs (HDAC1-3) can remove histone acetylation from the same sites as Sirt6 (*e.g.*, H3K9, H3K18, H3K27) and have been shown to exhibit histone delactylase activity as well (25, 43). Given this, we wanted to assess the relative contributions of Sirt6 and Zn^2+^-dependent HDACs to total Kac and Kla levels in our U2OS cell culture model. We treated the WT and S6KO U2OS cells with sodium L-lactate and with panobinostat, a nonselective inhibitor of the Zn^2+^-dependent HDACs (Classes I, II, IV) and again measured global histone acetylation and lactylation levels with the pan-antibodies (Fig. 5, *A*–*D*). Unsurprisingly, panobinostat increased the accumulation of total histone Kac (Fig. 5*D*, WT-p_1_ and KO- p_2_) and Kla (Fig. 5*B*, WT-p_3_ and KO- p_4_) in both cell lines. Sirt6 knockout did not affect the magnitude of the increase in bulk histone Kac caused by panobinostat treatment (Fig. 5*D*). However, in the presence of panobinostat (+lactate), Sirt6 knockout resulted in greater accumulation of total histone Kla (Fig. 5*B*, p_2_).

We next used the site-specific antibodies to analyze these same conditions (Fig. S4). As expected, panobinostat treatment elevated the acetylation and lactylation levels across the specific sites tested. For H3K9ac (Fig. S4*A*), we observed a significant difference between the Sirt6 WT and KO cells upon panobinostat treatment, demonstrating that when the Zn^2+^-dependent HDACs are restrained, the effect of Sirt6 KO on global H3K9ac levels is apparent. For H3K18la (Fig. S4*D*), we observed the same trends as for the pan-Kla data in Figure 5*B*. These data (Figs. 5*B* and S4*D*) suggest that the Zn^2+^-dependent HDACs cannot fully compensate for the loss of Sirt6 histone delactylation activity and *vice versa* in the U2OS cells. It suggests that Sirt6 and the Zn^2+^-dependent HDACs may differ in their activity at certain sites of histone lactylation and/or act at different loci within the genome. There is also likely compensation from Sirt1-3 that we did not examine in our experiments.

## Discussion

Previous studies seemed to “rule out” Sirt6 as a histone delactylase based on *in vitro* assays. However, these assays either used non-histone peptide substrates (44) or relied on using nicotinamide as a nonselective sirtuin inhibitor (43). Biochemical data indicated the existence of Sirt6-DNA and Sirt6-acidic patch interactions that are important for binding of Sirt6 to substrates and demonstrated that Sirt6 is much more active on nucleosome substrates than histone peptides (46, 47, 49), and recent cryo-electron microscopy studies confirmed these biochemical data (52, 54). Using nucleosome substrates, we detected and characterized the delactylase activity of Sirt6. We determined the kinetic parameters of Sirt6 for various PTM substrates, finding that the rate of Sirt6-catalyzed delactylation is similar to the rate of deacetylation. Another recent *in vitro* kinetics analysis of Sirt6 deacetylation and delactylation at H3K9 observed 5-fold faster removal for H3K9ac *versus* H3K9la mononucleosomes (56). Those assays were performed in a different buffer than ours (*i.e.*, we included NaCl and Mg^2+^) and used the 147-bp Widom 601 DNA, while we used 177-bp DNA that included extensions (*i.e.*, 15 bp-Widom 601 147 bp-15 bp). These differences are important due to their impact on Sirt6’s interactions with the nucleosome. In Wang *et al.*, the K_M,app_ measured for Sirt6 deacetylation of H3K9ac nucleosomes (147-bp DNA, no NaCl or Mg^2+^) was 17 nM, and the measured reaction velocity was much faster than ours (52). Conversely, our K_M,app_ values are in line with the magnitude of the K_d_ values (∼100 nM) reported by Chio *et al.* for Sirt6 binding to a nucleosome with a single 26-bp DNA extension in buffer with NaCl and Mg^2+^ (54). Furthermore, Chio *et al.* obtained a high-quality cryo-EM structure of nucleosome-bound Sirt6 from the 173-bp DNA, but not from 147-bp DNA, and posited that Sirt6 is more stable (*i.e.*, longer residence time) on a nucleosome with at least one DNA extension. Altogether, these observations point to potential differences in Sirt6’s binding kinetics on nucleosomes with (slower on/off) or without (faster on/off) DNA extension/s (and perhaps influenced also by NaCl and/or Mg^2+^). Additionally, while our study and Wang *et al.* (56) both used full-length human SIRT6, we used different purification methods, which could influence the enzyme’s activity as well.

Additionally, our data indicated that Sirt6 delactylates nucleosomes in cell culture at the H3K9 and H3K18 positions and that this delactylase behavior is additive with the delactylase behavior of the Zn^2+^-dependent HDACs. Since our data indicate that panobinostat (and thus Zn^2+^-dependent HDACs) also affects Kla levels at H3K9 and H3K18, it is likely that the Zn^2+^-dependent HDACs and Sirt6 are delactylating at different genomic loci in these cells, but further experiments will be necessary to test this hypothesis. While we only tested a subset of Kla sites in our study, Sirt6 can likely delactylate at the same sites that it deacetylates (46, 47, 48, 49, 50, 51, 56). Many of our cell culture experiments were carried out with the addition of 25 mM sodium L-lactate to the media. While this is well above the normal human serum level of lactate (1.5–3 mM), lactate concentrations from 10 to 50 mM are known to occur in inflammation and cancers (57, 58), and many of the phenotypes and diseases in which histone lactylation have been studied are closely connected to inflammation or cancer development and progression. Given this information, the lactate supplementation paradigm is a useful model to study effects of or regulators of histone lactylation. Finally, our data are consistent with foundational literature that identified the deacetylase behavior of Sirt6 in that decreases in global levels of histone acetylation, such as H3K9ac, are apparent in a range of different cell lines upon Sirt6 overexpression (47, 48, 59, 60). However, Sirt6 knockdown does not necessarily lead to global increases in histone acetylation in all cell types and was not observed upon Sirt6 knockdown in U2OS cells previously (48). Indeed, we did not observe higher total histone Kac upon Sirt6 KO (Fig. 4*E*), but when Zn^2+^-dependent HDACs were inhibited, we did see an increase in H3K9ac upon Sirt6 KO, supporting the hypothesis that there are compensation effects when looking at global histone PTM levels, even at a specific site.

Sirt6 has several experimentally demonstrated enzymatic functions including deacetylation, fatty deacylation, and ADP-ribosylation (61, 62, 63, 64). These enzymatic activities ultimately affect a variety of biological processes. Sirt6 is known to regulate metabolic homeostasis, and it has been implicated in regulating DNA repair processes and metabolic processes such as insulin secretion and ketogenesis (59, 63, 65, 66, 67, 68, 69). Sirt6 is specifically known to downregulate glycolytic activity in cells by downregulating the activity of Hif1α (70, 71). Given its role in regulating the expression of metabolic enzymes, it is plausible that Sirt6-catalyzed delactylation is part of a mechanism by which Sirt6 may regulate expression of metabolic genes. By identifying that Sirt6 is a histone delactylase, this work paves the way for further understanding of how Sirt6 and histone lactylation regulate metabolism.

## Experimental procedures

### Materials

All salt, buffers, and other chemicals were obtained from Fisher Scientific unless otherwise noted. All reagents used for solid-phase peptide synthesis (SPPS) were obtained from ChemImpex International unless otherwise noted. Other reagents for biochemical assays, cloning, and protein purification are listed in Table 1. Antibodies (with dilutions used) are listed in Table 2. The Sirt6 plasmid for *E. coli* expression was described in a previous study (72). SIRT7.4 was a gift from John Denu (Addgene plasmid # 13740; http://n2t.net/addgene:13740; RRID:Addgene_13740) (73). The histone plasmids were described in a previous study (74). pLJM1 was a gift from David Sabatini (Addgene plasmid # 19319) (75). pSpCas9(BB)-2A-Puro (PX459) V2.0 was a gift from Feng Zhang (Addgene plasmid # 62988; http://n2t.net/addgene:62988; RRID:Addgene_62988) (76). The HEK-293T cells were a gift from Jared Rutter. The U2OS cells were a gift from Glen Liszczak.

**Table 1:** Materials

Reagent	Manufacturer	Catalog No.
Q5 High-Fidelity DNA Polymerase	New England Biolabs (NEB)	M0491
Dpn1	New England Biolabs (NEB)	R0176
Bbs1	New England Biolabs (NEB)	R0539
T4 PNK	New England Biolabs (NEB)	M0201
T4 DNA ligase	New England Biolabs (NEB)	M0202
Micrococcal Nuclease	New England Biolabs (NEB)	M0247
6× NEB purple DNA loading dye	New England Biolabs (NEB)	B7025
100 bp ladder	New England Biolabs (NEB)	N0551
DH5α Mach1 competent cells	Invitrogen	C862003
Rosetta(DE3) competent cells	Sigma	70954
QG buffer	Qiagen	19063
Miniprep buffers and columns	Qiagen	27104
Protease inhibitor mini tablets	Pierce	A32953
High affinity Ni-charged resin	GenScript	L00223
HiLoad Superdex 16/600 200 pg column	Cytiva	28989335
Superdex 200 Increase 10/300 GL	Cytiva	28990944
Dialysis Tubing	ThermoFisher	88244
XBridge BEH C18 columns	Waters	186003624, 186008193, 186003673
NAD^+^	New England Biolabs (NEB)	B9007S
Revert 700 total protein stain	LI-COR	926-11010
MES 20× buffer	Bio-Rad	1610789
All blue ladder	Bio-Rad	1610373
Dual color ladder	Bio-Rad	1610374
PVDF membrane	Bio-Rad	1620177
Sodium L-Lactate	Thermo	AAL1450006
Sodium Oxamate	Thermo	A16532.06
Panobinostat	SelleckChem	LBH589
Rotenone	MP Biomedicals	150154

**Table 2:** Antibodies

Antibody	Dilution	Manufacturer	Catalog No.
Mouse anti-H3	1:2000	Cell Signaling Technologies	3638
Rabbit anti-L-Lactyl Lysine (pan Kla)	1:1000	PTM BIO	PTM-1401RM
Rabbit anti-acetyllysine (pan Kac)	1:1000	PTM BIO	PTM-105RM
Rabbit anti-H3K9ac	1:1000	Cell Signaling Technologies (CST)	9649
Rabbit anti-H3K9la	1:1000	PTM BIO	PTM-1419RM
Rabbit anti-H3K18ac	1:1000	Cell Signaling Technologies (CST)	9675
Rabbit anti-H3K18la	1:1000	PTM BIO	PTM-1406RM
Rabbit anti-H3K56ac	1:1000	Cell Signaling Technologies (CST)	4243
Rabbit anti-H3K56la	1:500	PTM BIO	PTM-1421RM
Rabbit anti-H4K12la	1:1000	PTM BIO	PTM-1411RM
Rabbit anti-Sirt6	1:1000	Cell Signaling Technologies (CST)	12486
Rabbit anti-Sirt7	1:1000	Cell Signaling Technologies (CST)	5360
Mouse anti-β-actin	1:1000	Cell Signaling Technologies (CST)	3700
IRDye 680CW goat anti-mouse	1:10,000	LI-COR	926-68070
IRDye 800CW goat anti-rabbit	1:15,000	LI-COR	926-32211

### Methods

#### Cloning

Q5 High-Fidelity DNA Polymerase (NEB) was used for all PCR amplification steps. All primers were obtained from the DNA/Peptide Core at the University of Utah. PCR products were treated with Dpn1 (NEB), and linear products were purified *via* a 1% agarose gel, extracted with QG buffer (Qiagen), and purified by spin column (Qiagen). NEBuilder HiFi DNA Assembly Master Mix (NEB) was used as described by the manufacturer. For blunt-end ligations, T4 PNK (NEB) and T4 DNA ligase (NEB) were used as indicated by the manufacturer. Reactions were transformed into DH5α Mach1 *E. coli* cells (Invitrogen) and plated on antibiotic-containing agar to select colonies for sequence verification. Plasmids were isolated from liquid cultures using Qiagen Miniprep buffers and spin columns as directed by the manufacturer. All plasmids were sequence verified by GENEWIZ (Aventa Life Sciences). Amino acid sequences are listed in the Supporting Information.

The pET28a-LIC-6x-His-SUMO-Sirt6 was cloned as described previously (72). The pQE-80-6xHis-Sirt7(Δexon2) was Addgene plasmid #13740. The missing exon two was synthesized as a GeneBlock (IDT) and cloned into the Sirt7 gene. A SUMO tag was also added between the 6xHis and the Sirt7 to yield pQE-80-6xHis-SUMO-Sirt7. For mammalian cell overexpression, the full-length, human Sirt6 sequence with a C-terminal FLAG tag was cloned into a pLJM1 mammalian expression vector with a CMV promoter (“pLJM1-Sirt6-3xFLAG”).,

For the human cell knockouts, the following sgRNA sequences were cloned into the pSpCas9(BB)-2A-Puro (PX459) V2.0 vector:

Sirt6: sgRNA1- CCTGAAGTCGGGGATGCCAG (77), sgRNA2- TACGTCCGAGACACAGTCGT (78).

Sirt7: sgRNA1- CGTTACCAGGTCCGCGCTCT, sgRNA2- GCTTCAGGCCCTCGCGCCGC, sgRNA3- GGCCCTGCAGCTCCGTTACC (79).

The oligonucleotide design and cloning were performed according to the protocol provided by the Zhang lab on the Addgene #62988 website. Briefly, the oligonucleotides were annealed and phosphorylated using T4 PNK (NEB) following the manufacturer’s protocol. 1 μg of the PX330 Cas9 plasmid was digested with *Bbs*I (NEB) following the manufacturer’s protocol and purified by agarose gel. Digested plasmid and phosphorylated/annealed oligo inserts were ligated using the T4 DNA Ligase (NEB) according to the manufacturer’s directions. The resulting plasmids were transformed into chemically competent DH5α *E. coli* cells. Colonies were selected, and plasmid identities were sequenced for verification.

#### Recombinant protein expression

Plasmids were transformed into BL21 Rosetta (DE3) *E. coli* cells (Millipore) and grown to OD 0.6 in LB Miller (Fisher), shaking at 180 rpm at 37 °C with the addition of the appropriate antibiotic for selection. The temperature was then lowered to 18 °C, and cells were induced by the addition of 0.5 mM IPTG (Fisher) and allowed to grow for 18 h before collection and storage at −80 °C.

#### Protein purifications

##### Histones

Full-length, human histones H2A, H2B, H3, and H4, as well as H3[ΔN, 1–14]A15C and H3[ΔN, 1–28]A29C were purified as described previously (74, 80).

##### Sirtuins

Full-length, human Sirt6 was expressed and purified as previously described (72). Briefly, cells containing the expressed 6xHis-SUMO-Sirt6 were lysed in lysis buffer (50 mM Tris, pH 7.5, 500 mM NaCl, 10 mM MgCl_2_, 5 mM BME, 1 mM PMSF). Sirt6 was purified using a nickel affinity resin (Genscript) and eluted in lysis buffer with 300 mM imidazole added. Ulp1 was added to the elution to cleave the 6x-His-SUMO tag, then the solution was dialyzed against lysis buffer with no imidazole or PMSF. The cleaved 6x-His-SUMO was removed using a reverse nickel affinity purification. The purified, cleaved Sirt6 was concentrated and analyzed on a HiLoad 16/600 Superdex 200 pg size exclusion column (Cytiva) and eluted in storage buffer (50 mM Tris, pH 7.5, 100 mM NaCl, 1 mM MgCl_2_., 10% glycerol, 2 mM DTT). Fractions containing Sirt6 were pooled, concentrated, and stored at −80 °C. Sirt7 was purified using the same protocol, except lysis and storage buffer did not contain MgCl_2_. A truncation product was detected, which could not able to be removed using SEC despite its apparently small size, but this did not seem to impact the activity of the isolated Sirt7. Purified proteins were analyzed *via* SDS-PAGE (Fig. S5*A*).

#### Histone H3 semisynthesis

The following histone H3 peptides were synthesized using a standard Fmoc-protected SPPS strategy as previously described (74, 80). The peptides were synthesized on a hydroxytrityl resin (Chemmatrix) functionalized with hydrazine.

H3(1–14, K9alloc): ARTKQTARK(alloc)STGGK-NHNH_2._

H3(1–28, K18alloc): ARTKQTARKSTGGKAPRK(alloc)QLATKAARKS-NHNH2.

After synthesis, the alloc protecting group was removed from the peptides on resin using palladium tetrakistriphenylphospine and dimethylbarbituric acid as previously described (74, 80). The K9ac and K18ac peptides were generated by coupling acetate to the deprotected ε-amine using 10% Acetic Anhydride/20% DIPEA in DMF. The K9lac and K18lac peptides were generated by coupling L-lactate to the deprotected ε-amine using 6 eq L-lactic acid, 5.5 eq PyAOP, and 12 eq of DIPEA in DMF. The peptides were then cleaved and fully deprotected using trifluoroacetic acid (95% TFA, 2.5% water, 2.5% triisopropylsilane).

The resulting hydrazide peptides were converted to thioester peptides following previously published protocols (74, 80). Briefly, each peptide was oxidized using NaNO_2_, then reacted with sodium mercaptoethane sulfonate (MESNa) to generate a thioester-containing peptide for native chemical ligation. The thioester peptides were purified using RP-HPLC (Agilent 1260) and characterized *via* LC-MS (Fig. S6). The MESNa peptides were then reacted with truncated histone proteins (H3[ΔN, 1–14]A15C for the H3K9ac and H3K9la peptides, H3[ΔN, 1–28]A29C for the H3K18ac and H3K18la peptides) following previously published protocols for native chemical ligation. Briefly, each peptide was reacted with the corresponding truncated histone and trifluoroethanethiol to generate the full-length histone, then the histones were subjected to radical desulfurization to yield the native H3A15 or H3A29. The resulting histones were purified *via* RP-HPLC (Agilent 1260 Infinity II) and characterized using LC-MS (Waters Acquity QDa and Waters Xevo G2-XS QTof) (Fig. S7).

#### Octamer/nucleosome formation

Wild type, H3K9ac, H3K9la, HK18ac, and K3K18la octamers were assembled by combining H3 (WT or modified) with H2A, H2B, and H4 and dialyzing as previously described (74, 80). Octamers were purified by size exclusion chromatography (Superdex 200 increase column, Cytiva). Mononucleosomes were assembled by adding ds601 DNA (sequence in Supporting Information) and dialyzing as previously described. The mononucleosomes were analyzed on a native 5% TBE gel to assess their quality (Fig. S5*C*).

#### Tissue culture

U2OS and HEK 293T cell lines were grown in Dulbecco’s modified Eagle’s media (DMEM, Gibco) supplemented with 10% fetal bovine serum (Gibco) and 1% penicillin/streptomycin (Gibco). Cells were routinely tested for *mycoplasma* contamination and periodically genotyped using STR analysis (ATCC). Cells were subcultured at 95% confluence; U2OS cells were routinely subcultured using a 1:8 dilution and HEK-293T cells were routinely subcultured using a 1:10 dilution. For all treatment experiments, cells were plated at least 24 h prior to treatment and allowed to grow to 80% confluence. Cells were subcultured for ≤ 12 passages. Cells were treated with sodium L-lactate (up to 100 mM), sodium oxamate (10 mM), or panobinostat (10 nM) for 24 h immediately prior to harvesting. Cells treated with rotenone (up to 15 nM) were treated for 4 h immediately prior to harvesting. Control cells were treated with vehicle (1% sterile PBS for sodium lactate and sodium oxamate, 0.5% DMSO for panobinostat and rotenone). All cell experiments were performed with three biological replicates. Cell lysis and histone acid extraction were performed using the EpiQuik total histone extraction kit (EpiGentek) according to manufacturer’s published protocols. Soluble lysates were obtained by preserving the supernatant from the initial lysis step of the histone extraction procedure (“Pre-lysis” according to the manufacturer’s nomenclature).

#### Nucleosome isolation

Approximately 3.0 × 10^7^ cells were lysed in hypotonic buffer (10 mM Tris, pH 7.5, 10 mM KCl, 1.5 mM MgCl_2_, 0.34 M sucrose, 10% glycerol, 1 mM DTT, 1× Pierce mini protease inhibitor tablet) with 0.1% NP-40 added (55). Cells were left on ice for 10 min, then pelleted at 1300 rcf for 5 min. The pellet was washed in hypotonic buffer, then resuspended in chromatin precipitation buffer (3 mM EDTA, 0.2 mM EGTA, 1 mM DTT, Pierce mini protease inhibitor tablet). The pellet was pelleted at 1700 rcf for 5 min, then washed with precipitation buffer again. The pellet was washed once with resuspension buffer (hypotonic buffer + 2 mM CaCl_2_). The suspension was sonicated with a microtip rod sonicator (40% amplitude, 15 s on, 45 s off, six cycles). Micrococcal nuclease was added (2000 u, 1 μl) and the mixture was incubated at 37 °C for 30 min. The reaction was quenched by adding EGTA to 10 mM. The suspension was then pelleted at 17,000 rcf for 5 min, and the nucleosomes were collected in the supernatant. These nucleosomes were also analyzed on a native 5% TBE gel to assess their size and quality (Fig. S5*C*)

#### CRISPR-Cas9 knockouts

For the Sirt6 knockout, separate 10-cm plates of U2OS cells were transfected with each Cas9/sgRNA plasmid. For the Sirt7 knockout, one 10-cm plate was transfected with the pooled sgRNA plasmids. Cells were at 90% confluence at transfection. Cells were transfected using Lipofectamine 3000 (Invitrogen) in Optimem (Gibco) following the manufacturer’s protocol. Cells received 15 μg total DNA per 10-cm plate. Cells were left in Lipofectamine 3000/DNA/Optimem for 6 h, then recovered in antibiotic-free complete DMEM. Beginning at 48 h post-transfection, the cells were selected using puromycin (1 μg/ml) in complete media.

For Sirt7 KO cells, the selection was continued for 96 h. The knockout was confirmed in the polyclonal cells by Western blot (Fig. S5*B*). For Sirt6 KO cells, after a 48-h selection, the cells were plated by dilution in 96-well plates to yield single colonies in DMEM without any puromycin. The single colonies were expanded and screened for Sirt6 KO by Western blot. All experiments were performed from one clonal cell line (designated ‘A6’). Verification of Sirt6 knockout in this clone is in Figure 4*B*.

#### Sirt6 overexpression

Wild-type and S6KO U2OS cells were plated in a 6-well plate at 2.0 × 10^5^ cells per well. Once cells reached 90% confluence, cells were transfected with the Sirt6 plasmid using Lipofectamine 3000 (Invitrogen) and Optimem (Gibco), following the manufacturer’s recommended protocol. Each well of cells received 1 μg of DNA. Cells were left in Optimem for 6 h, then recovered in antibiotic-free complete DMEM overnight. Cells were then incubated in complete DMEM for 48 h and harvested. Control cells (*i.e.* cells not receiving the overexpression plasmid) were subjected to the same treatment with lipofectamine, but with no addition of DNA, and received all the same washes and media changes as the treated cells.

#### *In vitro* sirtuin activity assays

For basic assays, testing whether Sirt6 or Sirt7 is active on a substrate, stock solutions of recombinant sirtuin (30 μM, 10×), NAD^+^ (10 mM, 10×), and 5× reaction buffer (250 mM Tris, pH 7.5, 100 mM NaCl, 10 mM MgCl_2_, 10 mM DTT) were prepared. The reaction buffer and stock solutions were diluted in milliQ water, and substrate nucleosome was added to a final concentration of 100 nM (for semisynthetic nucleosomes) or 500 nM (for nucleosomes isolated from cells). The reactions were performed in 30 μl volumes, and were incubated at 37 °C for 2 h, then quenched by addition of 10 μl 5× SDS loading buffer (250 mM Tris, pH 6.8, 10% w/v SDS, 30% v/v glycerol, bromophenol blue, 7.5% v/v BME). For kinetics experiments, the above assay was adjusted to be performed in a 96-well plate. A 5× master mix was prepared containing Sirt6 (500 nM), NAD^+^ (5 mM), and buffer components (250 mM Tris, pH 7.5, 100 mM NaCl, 10 mM MgCl_2_, 10 mM DTT). To 16 μl of the master mix was added substrate nucleosome to desired final concentration (25–150 nM) and milliQ water to reach 80 μl. Reaction times were calculated from the addition of substrate. Reactions were incubated at 37 °C and quenched by addition of 20 μl 5× SDS loading buffer at indicated time points. 96-well plate assays were loaded with milliQ water in the outer wells adjacent to the edge of the plate to avoid uneven evaporation of water from reactions. All assays were performed with three technical replicates.

#### Western blotting

Samples were denatured by boiling in standard SDS loading buffer (50 mM Tris, pH 6.8, 2% w/v SDS, 6% v/v glycerol, bromophenol blue, 1.5% v/v BME) for 1 min. Samples were analyzed on either a 12% Bis Tris PAGE gel (for histone analytes) or a 4 to 12% Bis Tris PAGE gel (for all other analytes) using 1× MES running buffer (Bio-Rad) at 180 V. Samples were transferred to a PVDF membrane using semi-dry transfer at 25 V for 30 min, then blocked with 3% dry milk in TBST (50 mM Tris, pH 7.5, 150 mM NaCl, 0.05% Tween-20) for 1 h. Membranes were washed 3× for 5 min with TBST, then incubated with primary antibody at the indicated dilution for 1 to 2 h at RT. Membranes were again washed 3× with TBST, then incubated with secondary antibody (1:10,000 for mouse secondary, 1:15,000 for rabbit secondary) for 1 h at RT. The membranes were again washed 2× with TBST and 1× with milliQ water for 5 min, then imaged using a LI-COR Odyssey imager. Cross-target reactivity profiles of site-specific antibodies from CST and PTM Bio were tested, along with the linear range of these antibodies (Fig. S8).

#### Software/data analysis

Quantitation of western blots was performed using densitometry tools in the ImageStudio software package (LI-COR). All quantified western blots were corrected by dividing the analyte signal by a loading control (typically histone H3 or Revert 700 fluorescent total protein stain). Following the correction, the data were either presented as a corrected fluorescence value (in a.u.) when all data were acquired on the same blot or normalized as a fold change from a control condition to allow better comparison between different blots/antibodies. All densitometry quantitations and corrections performed are available as Supplemental Data File 1. All plotting and statistical analysis were performed using GraphPad Prism. Linear fits were performed using simple linear regression (unconstrained) in GraphPad Prism. Kinetics analyses were performed by first corrected for loading within each replicate by dividing the loading control densitometry value from each lane by the mean of the loading control values for that replicate, then multiplying the densitometry value of the PTM signal by that correction ratio. The remaining data points were then converted to concentrations of substrate by first normalizing to the t = 0 condition, then multiplying by the starting concentration of substrate. The calculated concentration values were then fit using simple linear regression on the first three data points (R^2^ ≥ 0.8 for all fits) to find V_I, app_. The V_I_ values were then fitted using the Michaelis-Menten kinetics function in Prism (unconstrained) to calculate apparent kinetic parameters. Statistical comparisons were performed using Student’s *t* test analysis when comparing two groups, Student’s *t* test followed by multiple comparison correction when making multiple comparisons between the same two groups, and one-way ANOVA with an appropriate *post hoc* test (as noted in figure captions) for comparing more than two groups.

## Data availability

All data generated in this study are available in this article. Densitometry-based quantitation data are available as an Excel spreadsheet in Supplemental Data File 1. Uncropped versions of all images (including replicates) from this study are available in the Supporting Information.

## Supporting information

This article contains Supporting Information.

## Conflict of interest

The authors declare that they do not have any conflicts of interest with the content of this article.
