# Supplementary material for: Sirtuin 6 is a histone delactylase
Source: J Biol Chem. 2025 Oct 6;301(12):110795. doi: 10.1016/j.jbc.2025.110795 (PMC12664031; doi:10.1016/j.jbc.2025.110795)

Sirtuin 6 is a histone lysine delactylase (Supporting Information)

Supporting Information

Table S1: Michaelis-Menten Kinetics of Sirt6 on Singly-Modified Nucleosomes 2

Figure S1: *In vitro* site-specific deacylation patterns of Sirt6 3

Figure S2: *In vitro* Sirt6 and Sirt7 assays 4

Figure S3: Treatment of U2OS cells with lactate and oxamate 5

Figure S4: Site-specific deacylation patterns of Sirt6 and Class I HDACs 6

Figure S5: Characterization of recombinant proteins, CRISPR knockouts, and nucleosomes 8

Figure S6: MS and HPLC analysis of MESNa peptides for histone semisynthesis 9

Figure S7: MS and HPLC analysis of semisynthetic histones 10

Figure S8: Specificity and linearity testing of antibodies 11

Sequences 12

Uncropped Blots

- *In Vitro* Sirt6 delactylation assay (figure 1) 13
- *In Vitro* Sirt6 Kinetics assays (figures 1 and S2) 13
- Native nucleosome site-specific deacylation (figure S1) 18
- Lactate Treatment of U2OS cells (figure 2) 19
- Treatment of U2OS cells with Lactate and Oxamate (figure S3) 22
- Treatment of U2OS cells with Rotenone (figure 3) 23
- Site-specific PTM analysis of histones from U2OS cells (figures 4 and S4) 24
- Sirt6 Overexpression (figure 5) 27
- Treatment of U2OS cells with Lactate and Panobinostat (figures 6 and S4) 28
- *In Vitro* Sirt7 delactylation assay on H3K18 nucleosomes (figure S2) 29
- Quality Control Gels (figure S5) 30

Table S1: Michaelis-Menten Kinetics of Sirt6 on Singly Modified Mononucleosomes

PTM	$V_{max, app}$ (nmol*min^{-1})	$k_{cat, app}$ (min^{-1})	$K_{M, app}$ (nM)	R^2
H3K9Ac	1.4 ± 0.4	0.014 ± 0.004	110 ± 50	0.77
H3K18Ac	1.5 ± 0.3	0.015 ± 0.003	140 ± 50	0.88
H3K9La	1.7 ± 0.6	0.017 ± 0.006	220 ± 110	0.85
H3K18La	1.7 ± 0.7	0.017 ± 0.007	250 ± 150	0.86

Sirtuin 6 is a histone lysine delactylase (Supporting Information)

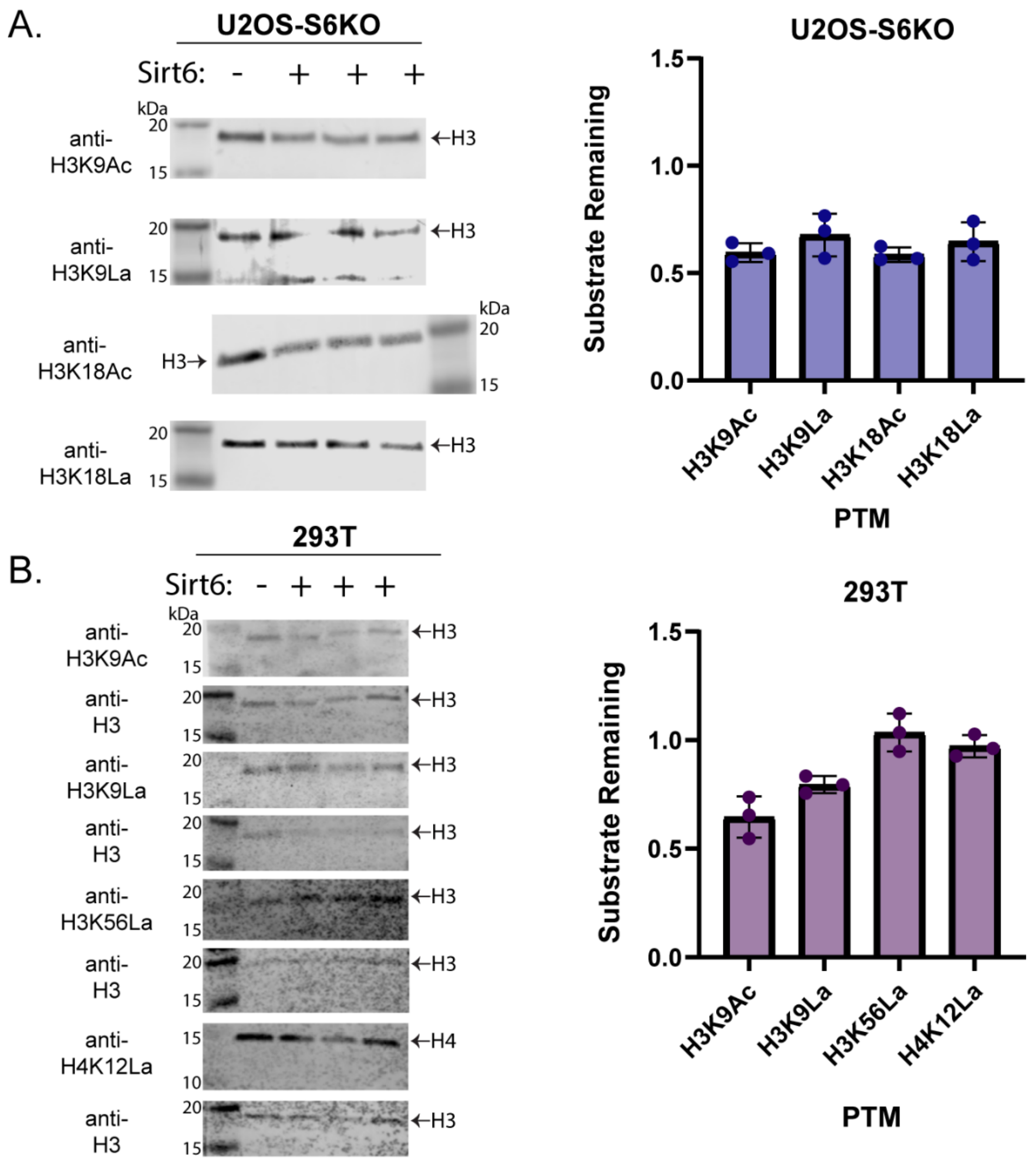

Figure S1: ***In vitro*** **site-specific deacylation patterns of Sirt6** (A) Nucleosomes isolated from U2OS-Sirt6 KO cells were incubated with Sirt6 and NAD^+, then levels of various acyl PTMs were measured via western blot. Data were quantified using densitometry and normalized to the “no Sirt6” signal. n=3 technical replicates, error plotted as S.D. (B) as in panel A, but for nucleosomes isolated from HEK-293T cells. The data were corrected based on the H3 loading control before being normalized to the “no Sirt6” condition.

Sirtuin 6 is a histone lysine delactylase (Supporting Information)

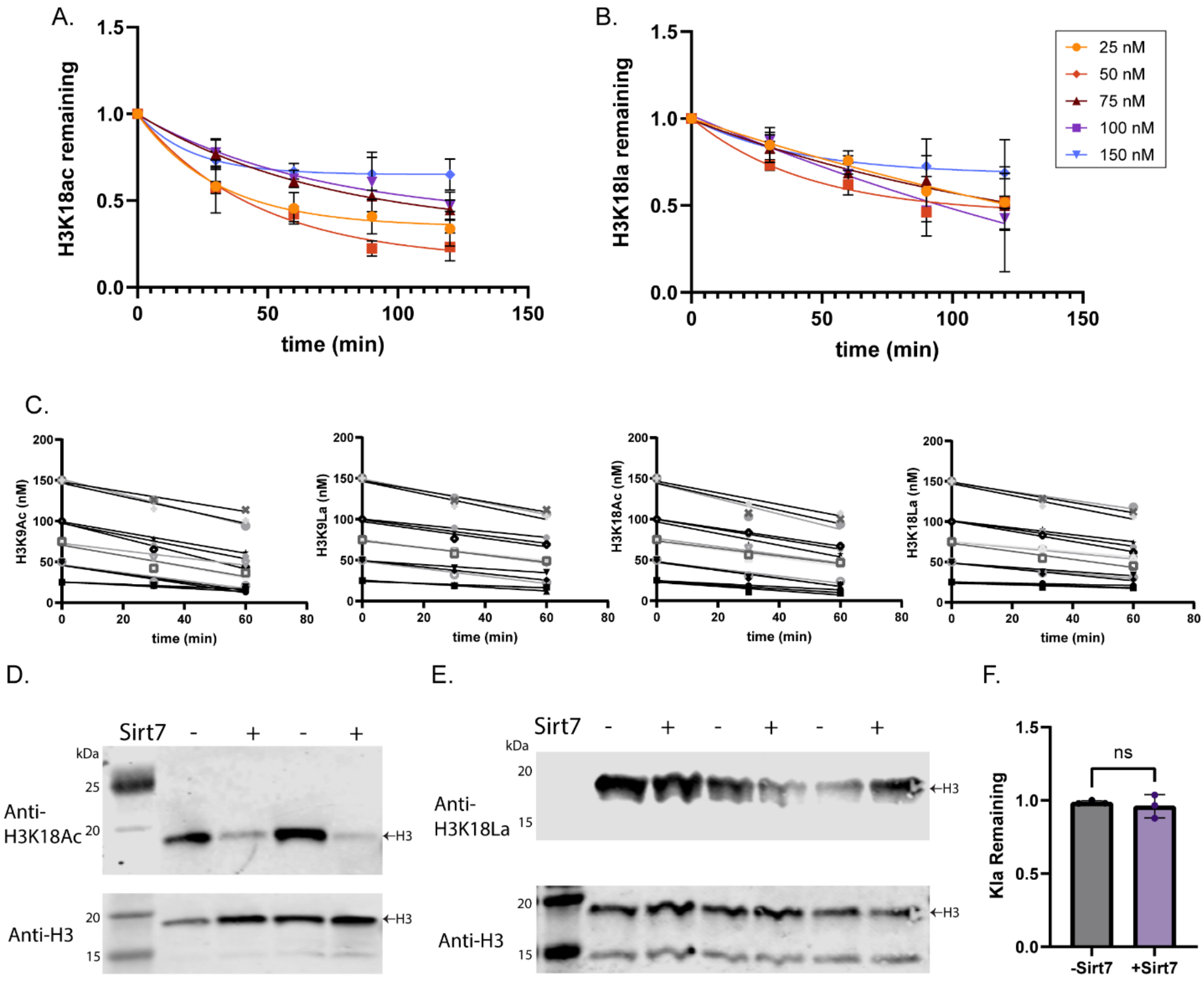

Figure S2: ***In Vitro* Sirt6 and Sirt7 assays**. (A) Sirt6 was incubated with varying concentrations of semisynthetic mononucleosome containing the H3K9ac PTM over 2 hours and the remaining substrate was measured via western blot. The data were quantified, corrected based on a loading control (histone H3 or a total protein stain), and are presented as a percentage of substrate signal remaining. The data were fit using a simple exponential decay model (y-int=1, plateau unconstrained). n=3 technical replicates for each concentration and time point, error plotted as S.D. (B) as in panel A, but for the H3K18ac PTM. (C) The linear regression analysis of the first hour of data collection from the kinetics data, used to determine V_i for each replicate of each condition. (D) Semisynthetic mononucleosomes (100 nM) containing the H3K18ac PTM were incubated with NAD^+ (1 mM) and with or without addition of Sirt7 (10 μM). Remaining H3K18ac was measured by western blot. Total protein stain (LiCor) was used as a loading control. (E) As in panel D, but for the H3K18la PTM. (F) Quantitation of data from panel E. Western blot data was quantified using densitometry, corrected based on the loading control, then each replicate was normalized as a fold change from the mean of the -Sirt7 condition. Statistical analysis was performed using Student'ts 2-tailed t-test, p=0.60. n=3 technical replicates, error plotted as S.D.

Sirtuin 6 is a histone lysine delactylase (Supporting Information)

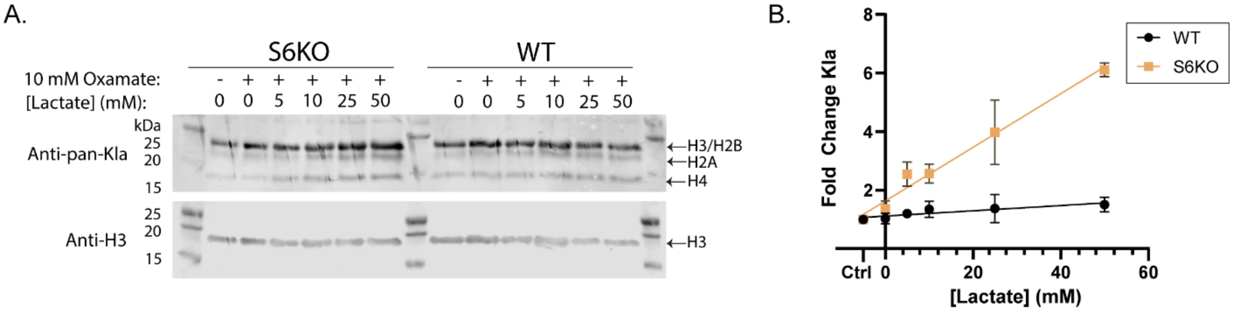

Figure S3: Treatment of U2OS cells with lactate and oxamate. (A) U2OS WT and Sirt6 knockout cells were treated with oxamate and titrated with sodium lactate as indicated. Histones were acid extracted from the cells and histone Kla levels were measured using a pan-lactyllysine antibody. (B) Quantitation of data from (a). Kla signal was corrected using the H3 loading control signal, then normalized as a fold change from the untreated condition, [lactate] = 0/[oxamate] = 0. n=3 biological replicates, error plotted as S.D. Slopes (starting at x=0) were compared using Welch's t-test, $p<0.0001$.

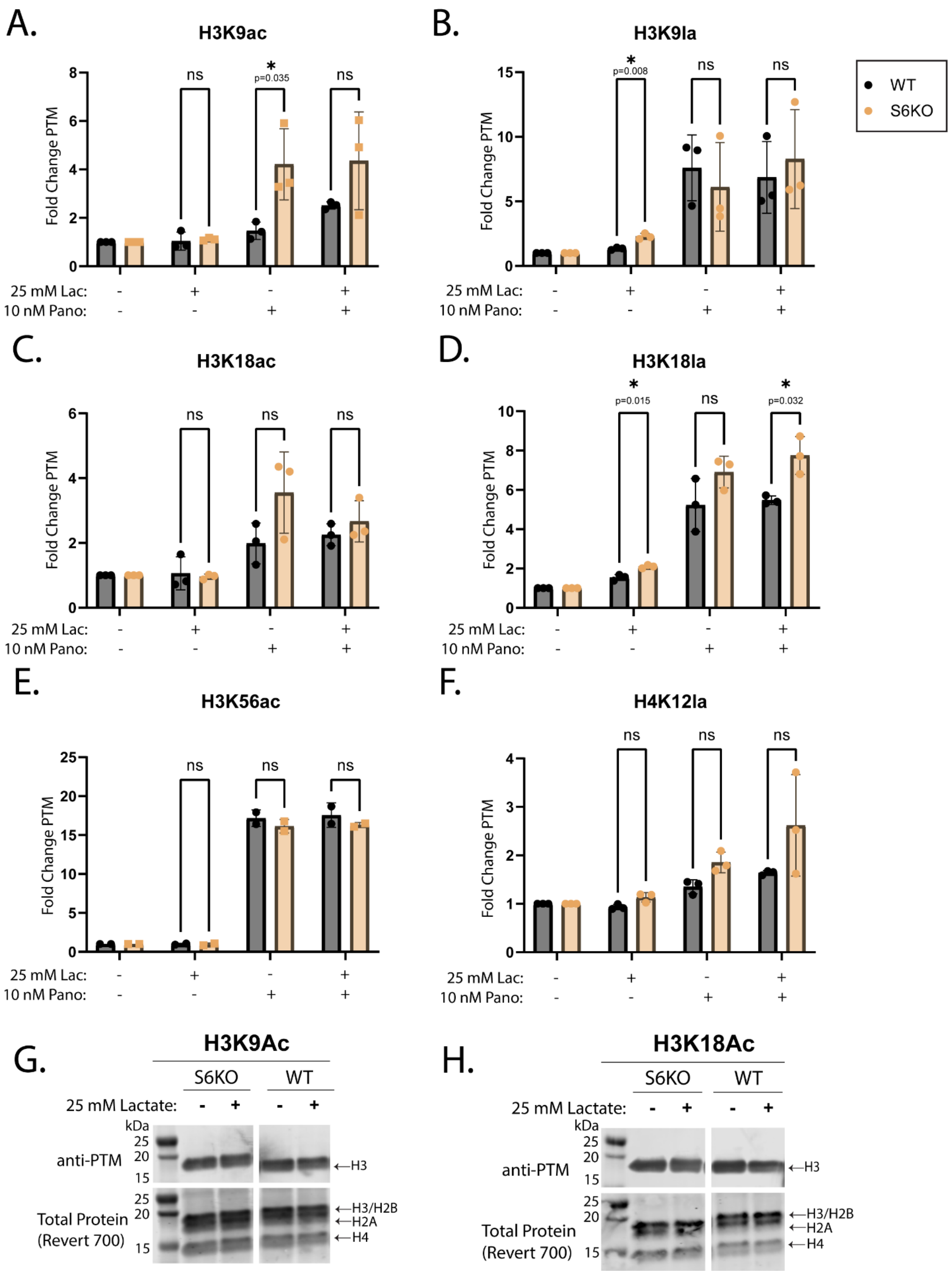
A.
H3K9ac
B.
H3K9la
WT
S6KO
C.
H3K18ac
D.
H3K18la
E.
H3K56ac
F.
H4K12la
Fold Change PTM
ns
*
p=0.035
p=0.008
p=0.015
p=0.032
25 mM Lac:
10 nM Pano:
G.
H3K9Ac
H.
H3K18Ac
S6KO
WT
25 mM Lactate:
kDa
anti-PTM
Total Protein
(Revert 700)
←H3
←H3/H2B
←H2A
←H4

Sirtuin 6 is a histone lysine delactylase (Supporting Information)

Figure S4: Site-specific deacylation patterns of Sirt6 and Class I HDACs. Wild-type and Sirt6-KO cells were treated with 10 nM Panobinostat, 25 mM sodium lactate, or both for 24 hours. Levels of PTMs on acid-extracted histones were measured using western blot using pan-PTM or site specific PTM antibodies (PTM Bio) as indicated. Western blot data was quantified using densitometry, corrected based on a loading control (histone H3 or total protein stain), and normalized as a fold change from the untreated condition within each cell line. Statistical analysis was performed using Student's 2-tailed t-test and corrected for multiple comparisons using the Holm-Šídák correction. For (A)-(D) and (F), n=3 biological replicates. For (E), n=2 biological replicates. Error is plotted as S.D. for all panels. Note: The same "+lactate/-pano" data in panels A, D, and F are also displayed in Figure 3. (G) Levels of H3K9ac on acid-extracted histones from wild type or Sirt6 knockout U2OS cells were measured using site-specific antibodies (CST and PTM Bio). Total protein was measured using a fluorescent total protein stain (LI-COR). (H) Levels of H3K18ac on acid-extracted histones from wild type or Sirt6 knockout U2OS cells were measured using site-specific antibodies (CST and PTM Bio). Total protein was measured using a fluorescent total protein stain (LI-COR).

Sirtuin 6 is a histone lysine delactylase (Supporting Information)

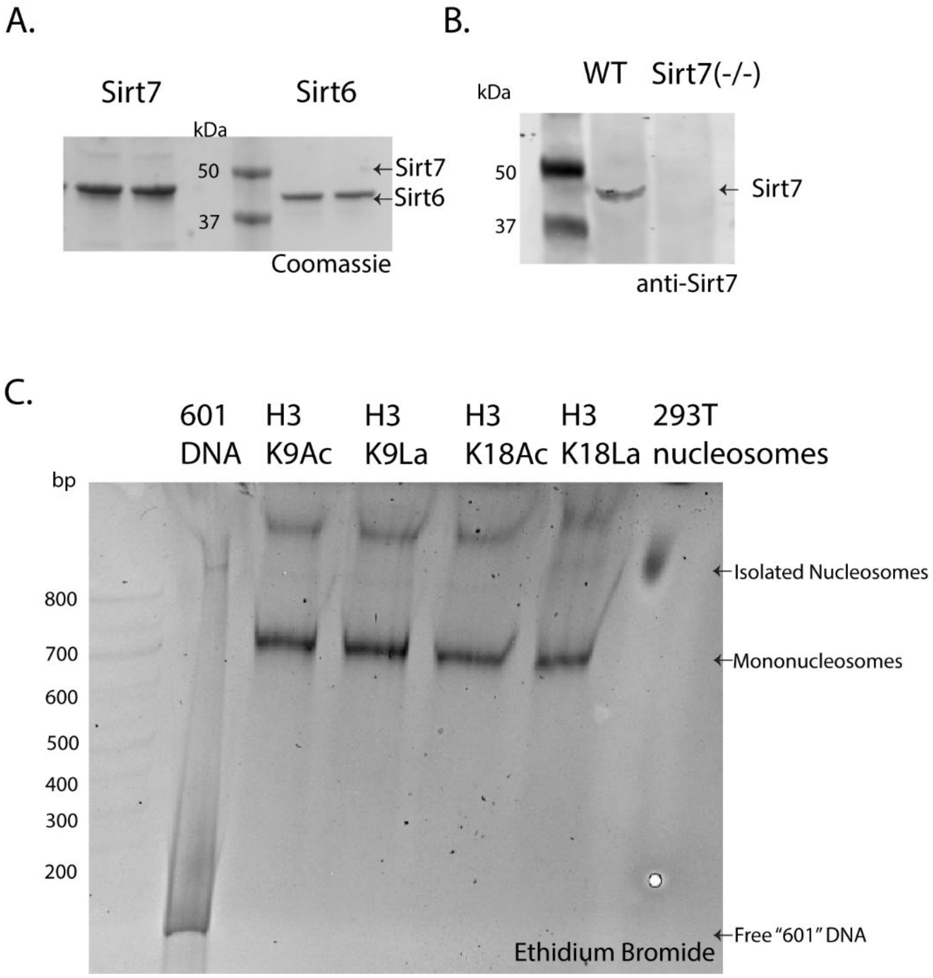

Figure S5: Characterization of recombinant proteins, CRISPR knockouts, and nucleosomes. (A) Coomassie stain of recombinant, full length, human Sirt6 and Sirt7 used in all *in vitro* assays. (B) Validation of Sirt7 knockout in the (-/-) Sirt7-KO cells generated using CRISPR-Cas9. Validation of Sirt6 knockout is in main-text figure 5. (C) EtBr-stained native TBE gel of all 4 semisynthetic nucleosomes and the nucleosomes isolated from 293T cells.

Sirtuin 6 is a histone lysine delactylase (Supporting Information)

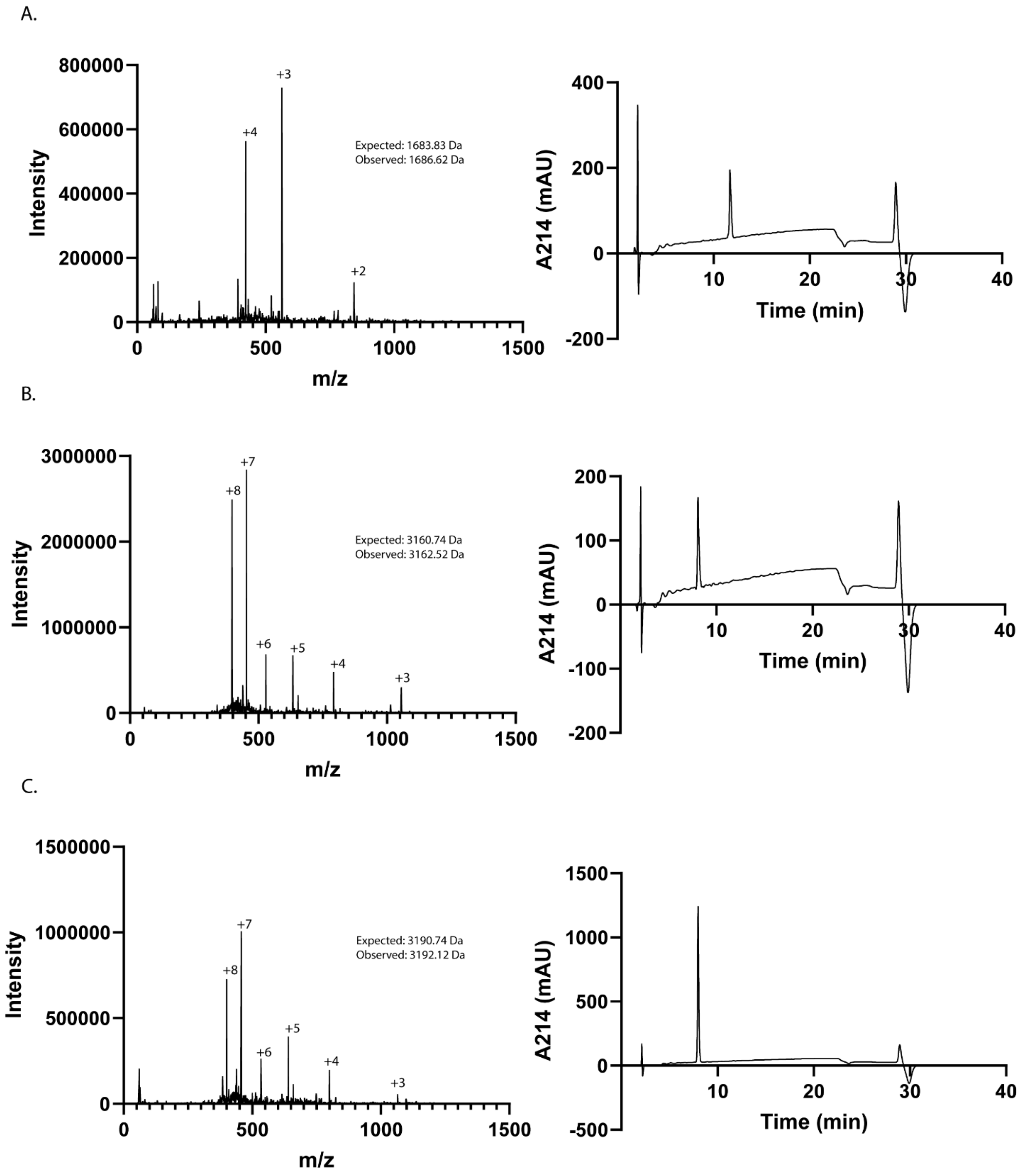

Figure S6: MS and HPLC analysis of MESNa peptides for histone semisynthesis. (A) LC-MS data (Waters QDa) and HPLC trace data (Waters XBridge BEH C18 analytical column) for H3K9la-MESNa peptide. (B) Data for H3K18ac-MESNa peptide. (C) Data for H3K18la-MESNa peptide. All HPLC traces collected using a 0-70% gradient of acetonitrile in water/0.1% TFA over 20 minutes.

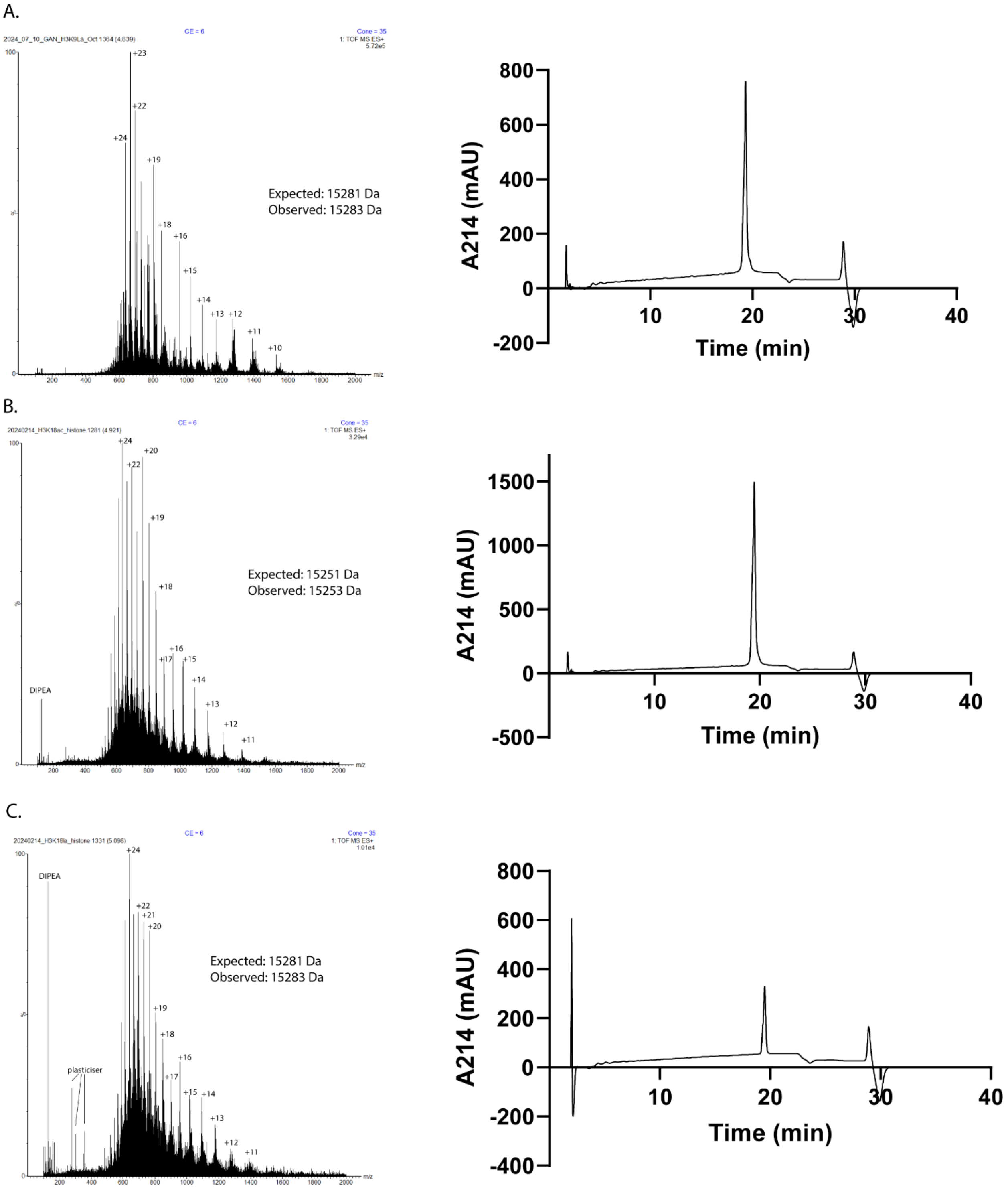

Figure S7: MS and HPLC analysis of semisynthetic histones. (A) High-resolution LC-MS data and HPLC trace for semisynthetic H3K9la histone. (B) Data for H3K18Ac histone. (C) Data for H3K18La histone. For all HPLC traces, separation was performed using a Waters BEH Xbridge C18 analytical column using a 0-70% gradient of acetonitrile in water/0.1% TFA over 20 min.

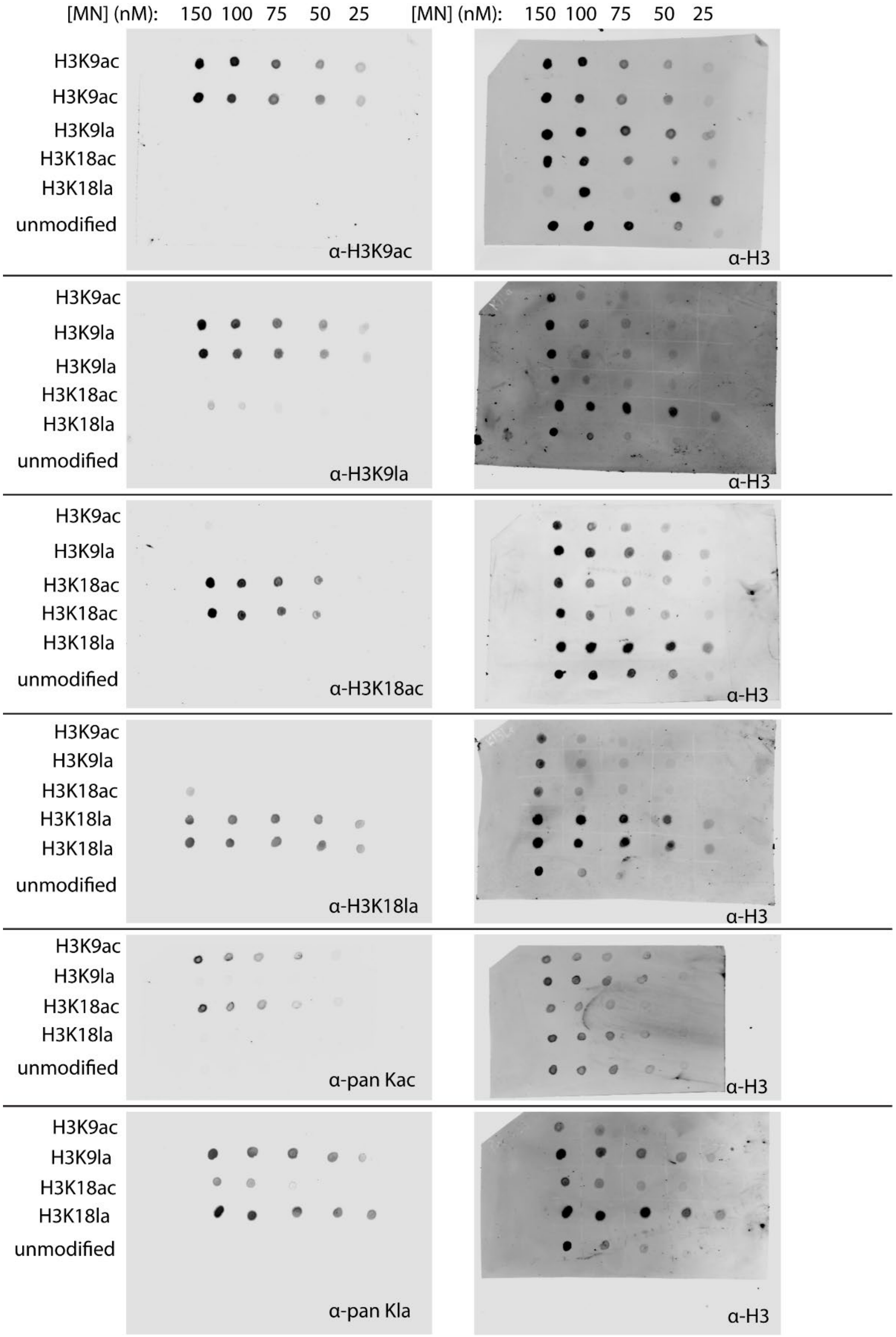

Figure S8: Specificity and linearity testing of antibodies. Modified and unmodified mononucleosomes were dot-blotted onto nitrocellulose at the indicated concentrations (2 µL/dot). The membranes were incubated with the indicated primary antibodies to test specificity and linearity of the antibodies within the concentration ranges used for our kinetics assays.

Sirtuin 6 is a histone lysine delactylase (Supporting Information)

Sequences

Key: **Bold = protein of interest**, underlined = SUMO tag

pET28a-LIC-6x-His-SUMO-Sirt6:

MGSSHHHHHHGSGLVPRGSASMSDSEVNQEAKPEVKPEVKPETHINLKVSDGSSEIFFKIKKTTPLRRLMEAFAKRQGKEMDSLRFLYDGIRIQADQTPEDLDMEDNDIIEAHREQIGG**SVNYAAGLSPYADKGKCGLPEIFDPPEELERKVWELARLVWQSSSVVFHTGAGISTASGIPDFRGPHGVWTMEERGLAPKFDTTFESARPTQTHMALVQLERVGLLRFLVSQNVDGLHVRSGFPRDKLAELHGNMFVEECAKCKTQYVRDTVVGTMGLKATGRLCTVAKARGLRACRGELRDTILDWEDSLPDRDLALADEASRNADLSITLGTSLQIRPSGNLPLATKRRGGRLVIVNLQPTKHDRHADLRIHGYVDEVMTRLMKHLGLEIPAWDGPRVLERALPPLPRPPTPKLEPKEESPTRINGSIPAGPKQEPCAQHNGSEPASPKRERPTSPAPHRPPKRVKAKAVPS** *

pQE-80-6xHis-SUMO-Sirt7:

MRGSHHHHHHGSASDSEVNQEAKPEVKPEVKPETHINLKVSDGSSEIFFKIKKTTPLRRLMEAFAKRQGKEMDSLRFLYDGIRIQADQTPEDLDMEDNDIIEAHREQIGG**AAGGLSRSERKAAERVRRLREEQQRERLRQVCDDPEELRGKVRGLASAVRNAKYLVVYTGAGISTAASIPDYRGPNGVWTLLQKGRSVSAADLSEAEPTLTHMSITRLHEQKLVQHVVSQNCDGLHLRSGLPRTAISELHGNMYIEVCTSCVPNREYVRVFDVTERTALHRHQTGRTCHKCGTQLRDTIVHFGERGTLGQPLNWEAATEAASRADTILCLGSSLKVLKKYPRLWCMTKPPSRRPKLYIVNLQWTPKDDWAALKLHGKCDDVMRLLMAELGLEIPAYSRWQDPIFSLATPLRAGEEGSHSRKSLCRSREEAPPGDRGAPLSSAPILGGWFGRGCTKRTKRKKVT**EFGGDYKDDDDK*

pLJM1-Sirt6-3xFLAG

M**SVNYAAGLSPYADKGKCGLPEIFDPPEELERKVWELARLVWQSSSVVFHTGAGISTASGIPDFRGPHGVWTMEERGLAPKFDTTFESARPTQTHMALVQLERVGLLRFLVSQNVDGLHVRSGFPRDKLAELHGNMFVEECAKCKTQYVRDTVVGTMGLKATGRLCTVAKARGLRACRGELRDTILDWEDSLPDRDLALADEASRNADLSITLGTSLQIRPSGNLPLATKRRGGRLVIVNLQPTKHDRHADLRIHGYVDEVMTRLMKHLGLEIPAWDGPRVLERALPPLPRPPTPKLEPKEESPTRINGSIPAGPKQEPCAQHNGSEPASPKRERPTSPAPHRPPKRVKAKAVPS**EFGGDYKDDDDKGGSDYKDDDDKGGSDYKDDDDK*

ds601 DNA:

Key: 601 sequence (147 bp) in bold

CTACTGGTACGGCAG**ACAGGATGTATATATCTGACACGTGCCTGGAGACTAGGGAGTAATCCCCTTGGCGGTTAAAACGCGGGGGACAGCGCGTACGTGCGTTTAAGCGGTGCTAGAGCTGTCTACGACCAATTGAGCGGCCTCGGCACCGGGATTCTCCAG**TATTCGAGGCCGTTC

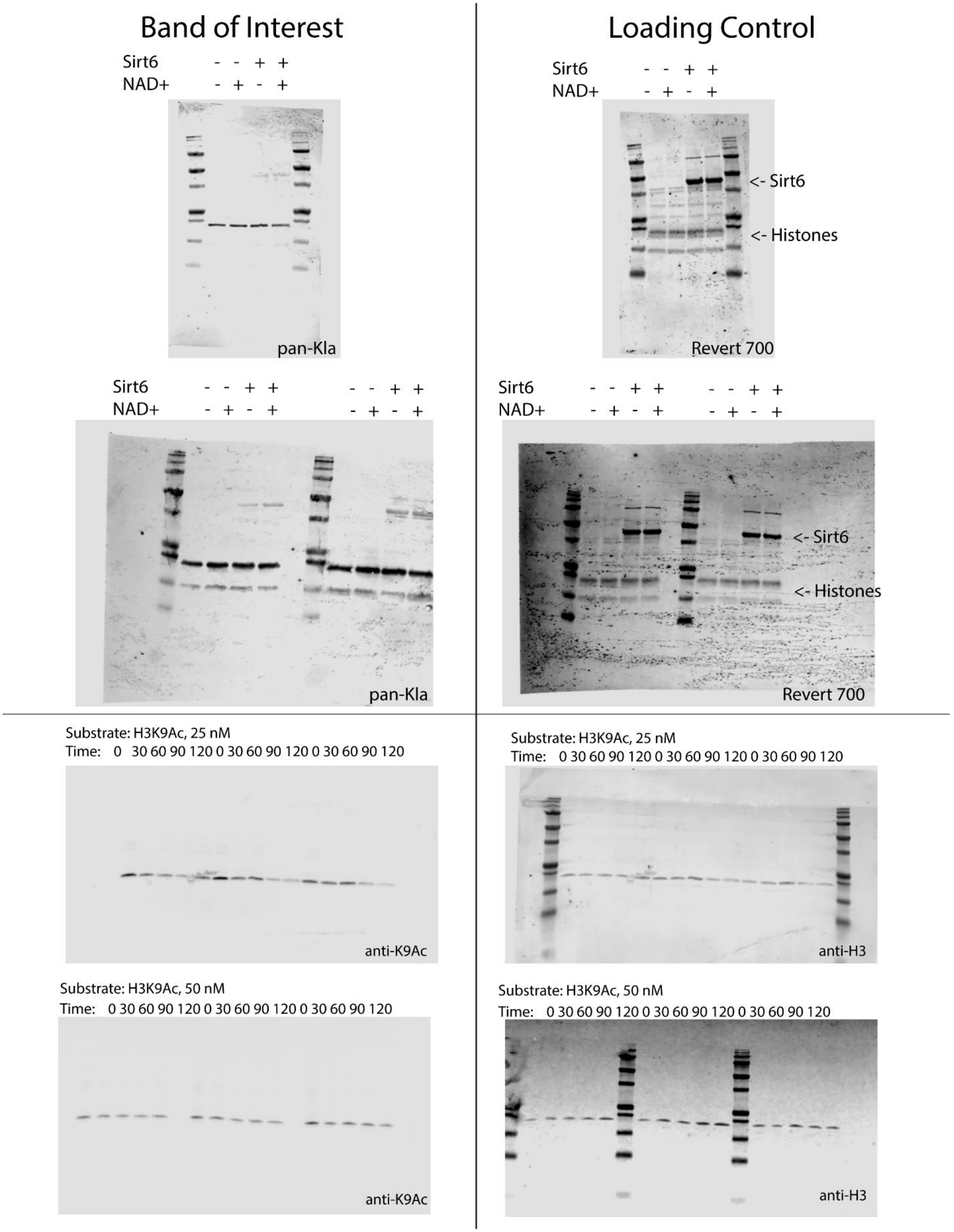
Band of Interest
Loading Control
Sirt6 - - + +
NAD+ - + - +
pan-Kla
<- Sirt6
<- Histones
Revert 700
Sirt6 - - + + - - + +
NAD+ - + - + - + - +
pan-Kla
<- Sirt6
<- Histones
Revert 700
Substrate: H3K9Ac, 25 nM
Time: 0 30 60 90 120 0 30 60 90 120 0 30 60 90 120
anti-K9Ac
Substrate: H3K9Ac, 25 nM
Time: 0 30 60 90 120 0 30 60 90 120 0 30 60 90 120
anti-H3
Substrate: H3K9Ac, 50 nM
Time: 0 30 60 90 120 0 30 60 90 120 0 30 60 90 120
anti-K9Ac
Substrate: H3K9Ac, 50 nM
Time: 0 30 60 90 120 0 30 60 90 120 0 30 60 90 120
anti-H3

Sirtuin 6 is a histone lysine delactylase (Supporting Information)

Band of Interest

Substrate: H3K9Ac, 75 nM
Time: 0 30 60 90 120 0 30 60 90 120 0 30 60 90 120

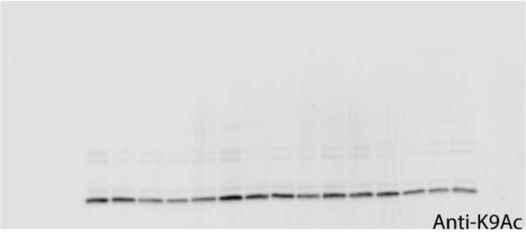

Anti-K9Ac

Substrate: H3K9Ac, 100 nM
Time: 0 30 60 90 120 0 30 60 90 120 0 30 60 90 120

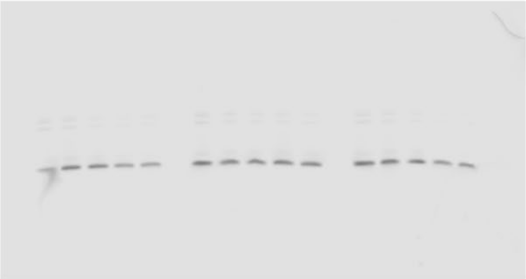

Anti-K9Ac

Substrate: H3K9Ac, 150 nM
Time: 0 30 60 90 120 0 30 60 90 120 0 30 60 90 120

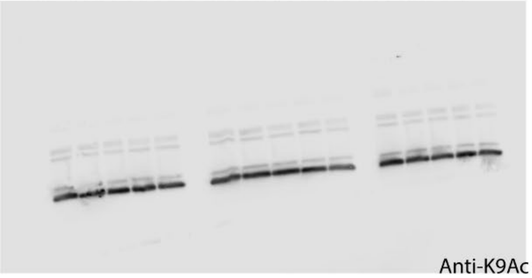

Anti-K9Ac

Substrate: H3K9La, 25 nM
Time: 0 30 60 90 120 0 30 60 90 120 0 30 60 90 120

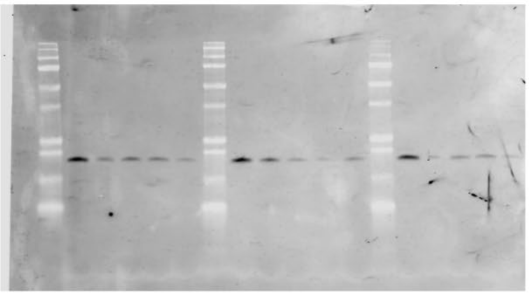

Anti-K9La

Loading Control

Substrate: H3K9Ac, 75 nM
Time: 0 30 60 90 120 0 30 60 90 120 0 30 60 90 120

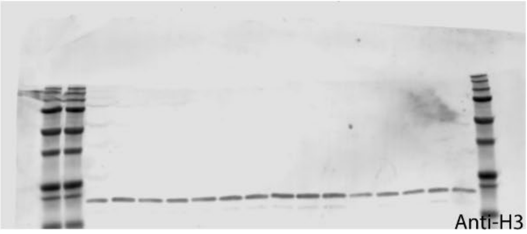

Anti-H3

Substrate: H3K9Ac, 100 nM
Time: 0 30 60 90 120 0 30 60 90 120 0 30 60 90 120

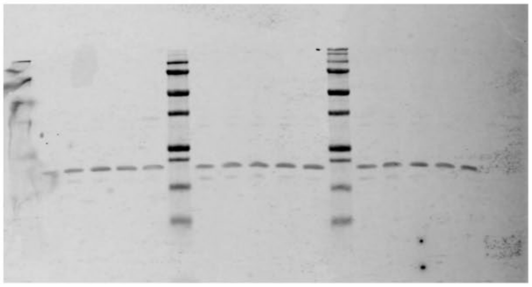

Anti-H3

Substrate: H3K9Ac, 150 nM
Time: 0 30 60 90 120 0 30 60 90 120 0 30 60 90 120

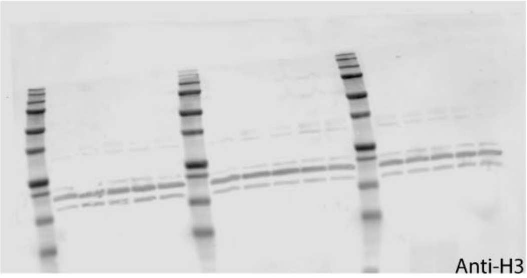

Anti-H3

Substrate: H3K9La, 25 nM
Time: 0 30 60 90 120 0 30 60 90 120 0 30 60 90 120

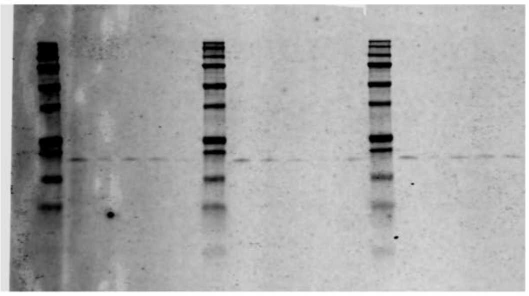

Revert 700

Band of Interest

Substrate: H3K9La, 50 nM
Time: 0 30 60 90 120 0 30 60 90 120 0 30 60 90 120

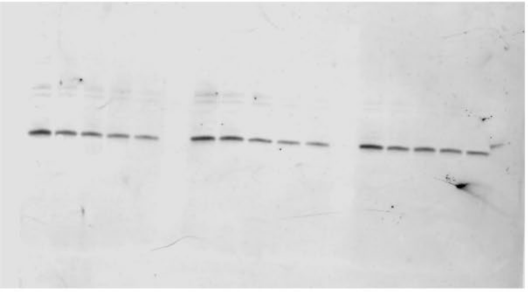

Anti-K9La

Substrate: H3K9La, 75 nM
Time: 0 30 60 90 120 0 30 60 90 120 0 30 60 90 120

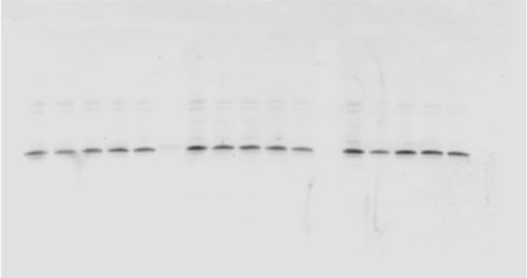

Anti-K9La

Substrate: H3K9La, 100 nM
Time: 0 30 60 90 120 0 30 60 90 120 0 30 60 90 120

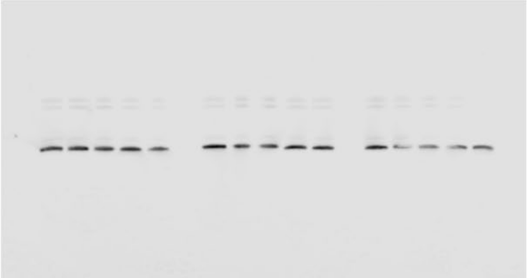

Anti-K9La

Substrate: H3K9La, 150 nM
Time: 0 30 60 90 120 0 30 60 90 120 0 30 60 90 120

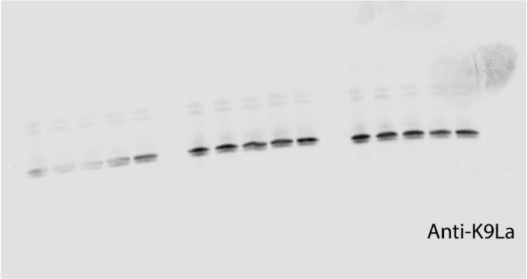

Loading Control

Substrate: H3K9La, 50 nM
Time: 0 30 60 90 120 0 30 60 90 120 0 30 60 90 120

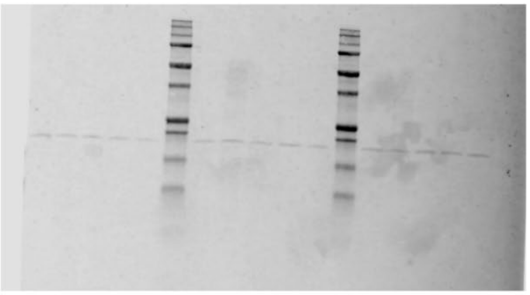

Revert 700

Substrate: H3K9La, 75 nM
Time: 0 30 60 90 120 0 30 60 90 120 0 30 60 90 120

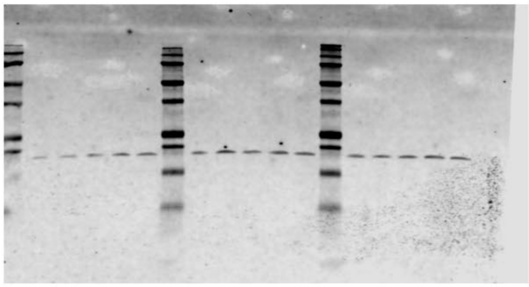

Revert 700

Substrate: H3K9La, 100 nM
Time: 0 30 60 90 120 0 30 60 90 120 0 30 60 90 120

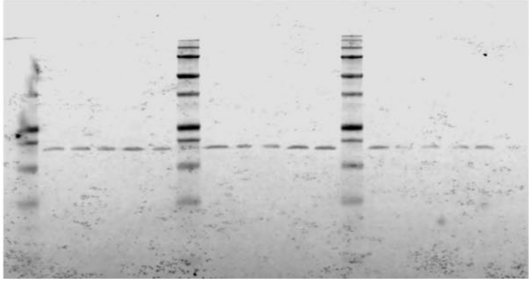

Revert 700

Substrate: H3K9La, 150 nM
Time: 0 30 60 90 120 0 30 60 90 120 0 30 60 90 120

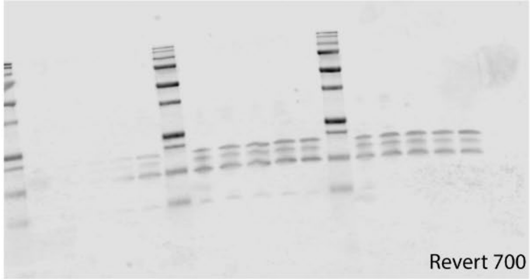

Band of Interest

Substrate: H3K18Ac, 25nM
Time: 0 30 60 90 120 0 30 60 90 120 0 30 60 90 120

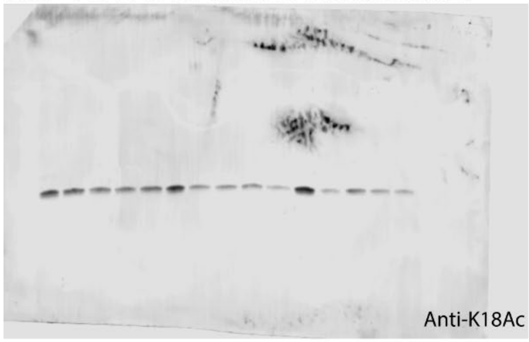

Substrate: H3K18Ac, 50 nM
Time: 0 30 60 90 120 0 30 60 90 120 0 30 60 90 120

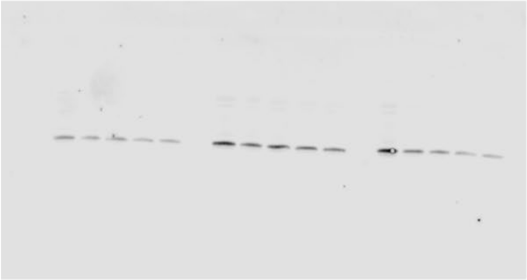

Anti-K18Ac

Substrate: H3K18Ac, 75 nM
Time: 0 30 60 90 120 0 30 60 90 120 0 30 60 90 120

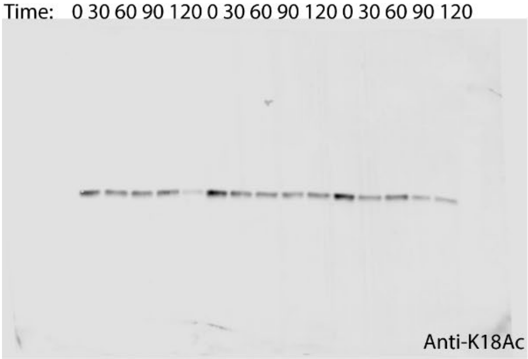

Substrate: H3K18Ac, 100 nM
Time: 0 30 60 90 120 0 30 60 90 120 0 30 60 90 120

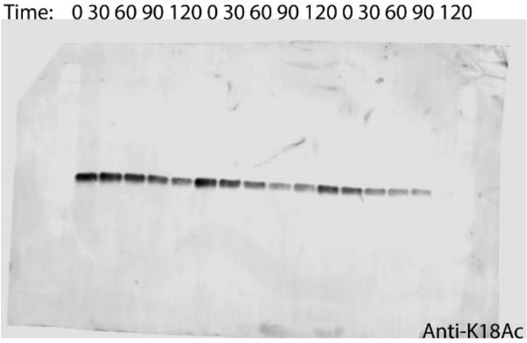

Loading Control

Substrate: H3K18Ac, 25nM
Time: 0 30 60 90 120 0 30 60 90 120 0 30 60 90 120

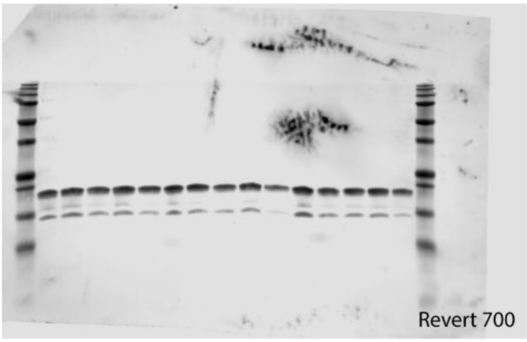

Substrate: H3K18Ac, 50 nM
Time: 0 30 60 90 120 0 30 60 90 120 0 30 60 90 120

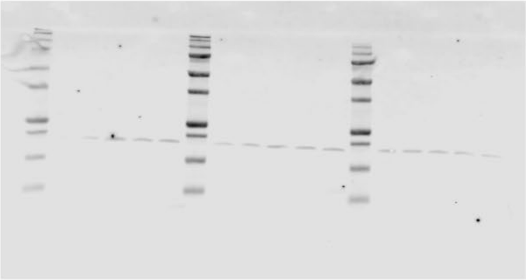

Revert 700

Substrate: H3K18Ac, 75nM
Time: 0 30 60 90 120 0 30 60 90 120 0 30 60 90 120

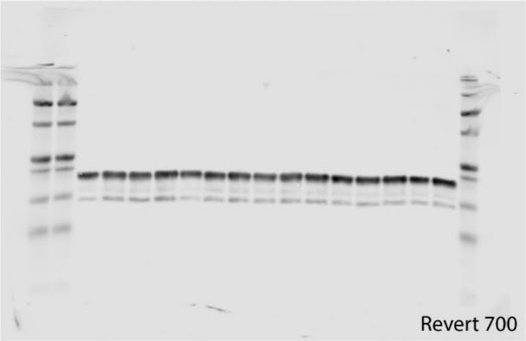

Substrate: H3K18Ac, 100nM
Time: 0 30 60 90 120 0 30 60 90 120 0 30 60 90 120

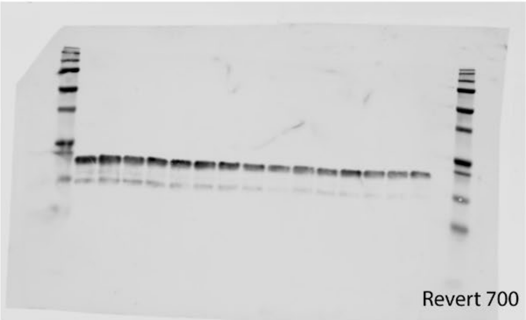

Band of Interest

Substrate: H3K18Ac, 150 nM
Time: 0 30 60 90 120 0 30 60 90 120 0 30 60 90 120

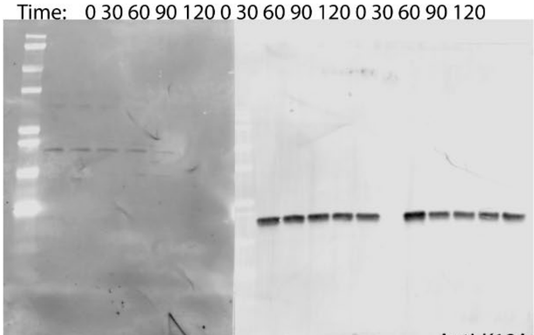

Anti-K18Ac

Substrate: H3K18La, 25 nM
Time: 0 30 60 90 120 0 30 60 90 120 0 30 60 90 120

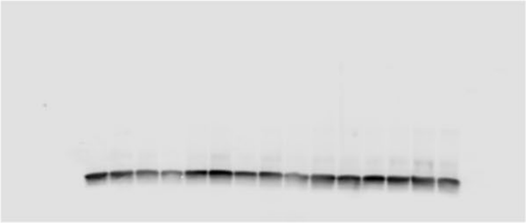

Anti-K18La

Substrate: H3K18La, 50 nM
Time: 0 30 60 90 120 0 30 60 90 120 0 30 60 90 120

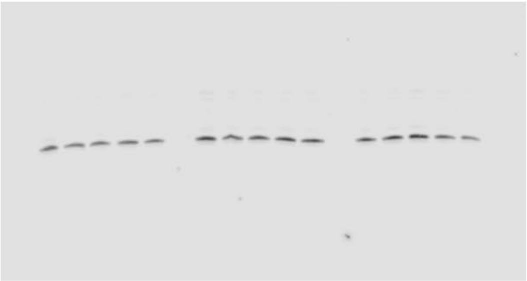

Anti-K18La

Substrate: H3K18La, 75 nM
Time: 0 30 60 90 120 0 30 60 90 120 0 30 60 90 120

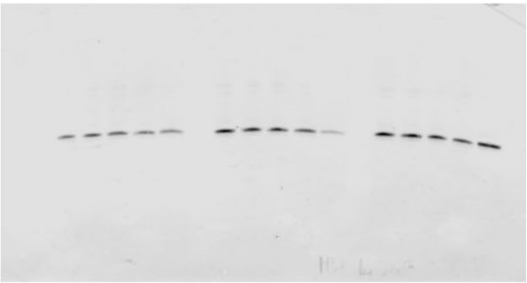

Anti-K18La

Loading Control

Substrate: H3K18Ac, 150 nM
Time: 0 30 60 90 120 0 30 60 90 120 0 30 60 90 120

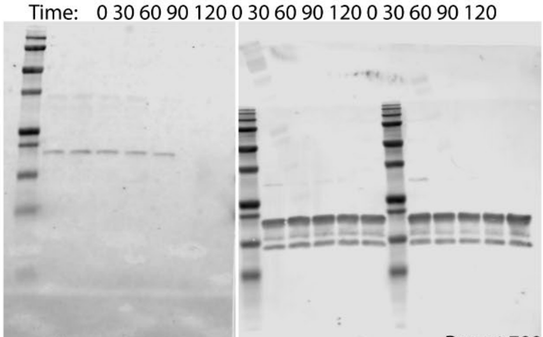

Revert 700

Substrate: H3K18La, 25 nM
Time: 0 30 60 90 120 0 30 60 90 120 0 30 60 90 120

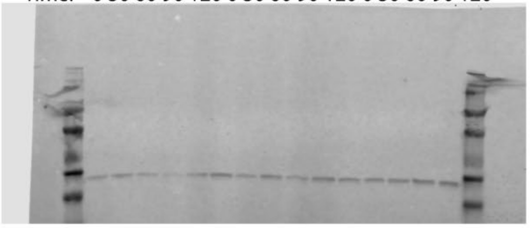

anti-H3

Substrate: H3K18La, 50 nM
Time: 0 30 60 90 120 0 30 60 90 120 0 30 60 90 120

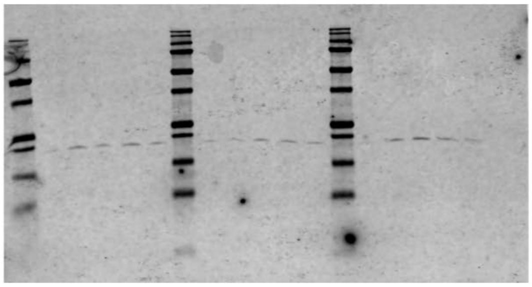

Revert 700

Substrate: H3K18La, 75 nM
Time: 0 30 60 90 120 0 30 60 90 120 0 30 60 90 120

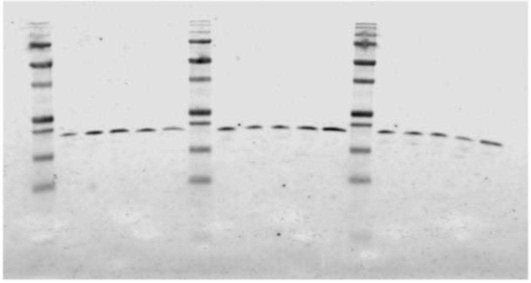

anti-H3

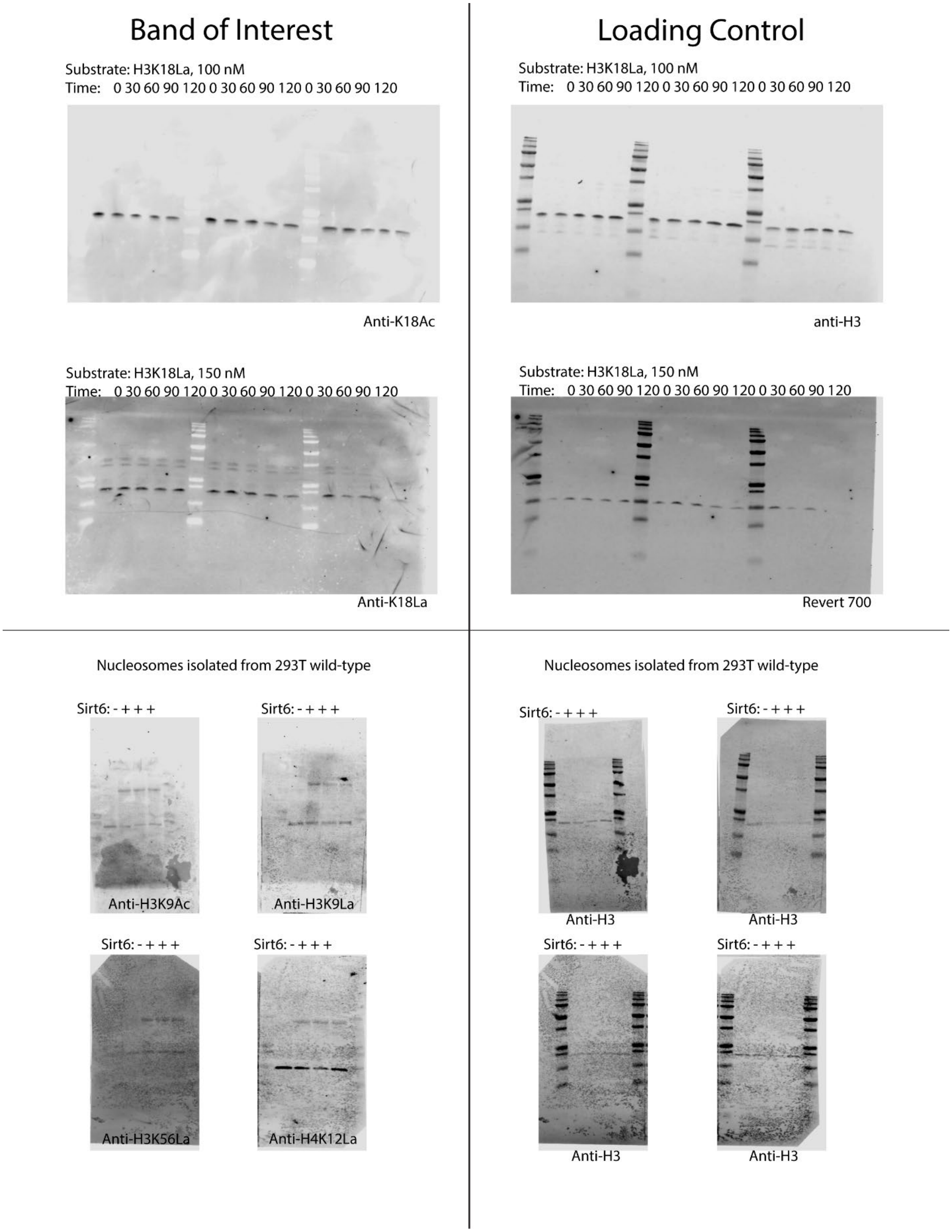
Band of Interest
Loading Control
Substrate: H3K18La, 100 nM
Time: 0 30 60 90 120 0 30 60 90 120 0 30 60 90 120
Anti-K18Ac
Substrate: H3K18La, 100 nM
Time: 0 30 60 90 120 0 30 60 90 120 0 30 60 90 120
anti-H3
Substrate: H3K18La, 150 nM
Time: 0 30 60 90 120 0 30 60 90 120 0 30 60 90 120
Anti-K18La
Substrate: H3K18La, 150 nM
Time: 0 30 60 90 120 0 30 60 90 120 0 30 60 90 120
Revert 700
Nucleosomes isolated from 293T wild-type
Sirt6: - + + +
Anti-H3K9Ac
Sirt6: - + + +
Anti-H3K9La
Sirt6: - + + +
Anti-H3K56La
Sirt6: - + + +
Anti-H4K12La
Nucleosomes isolated from 293T wild-type
Sirt6: - + + +
Anti-H3
Sirt6: - + + +
Anti-H3
Sirt6: - + + +
Anti-H3
Sirt6: - + + +
Anti-H3

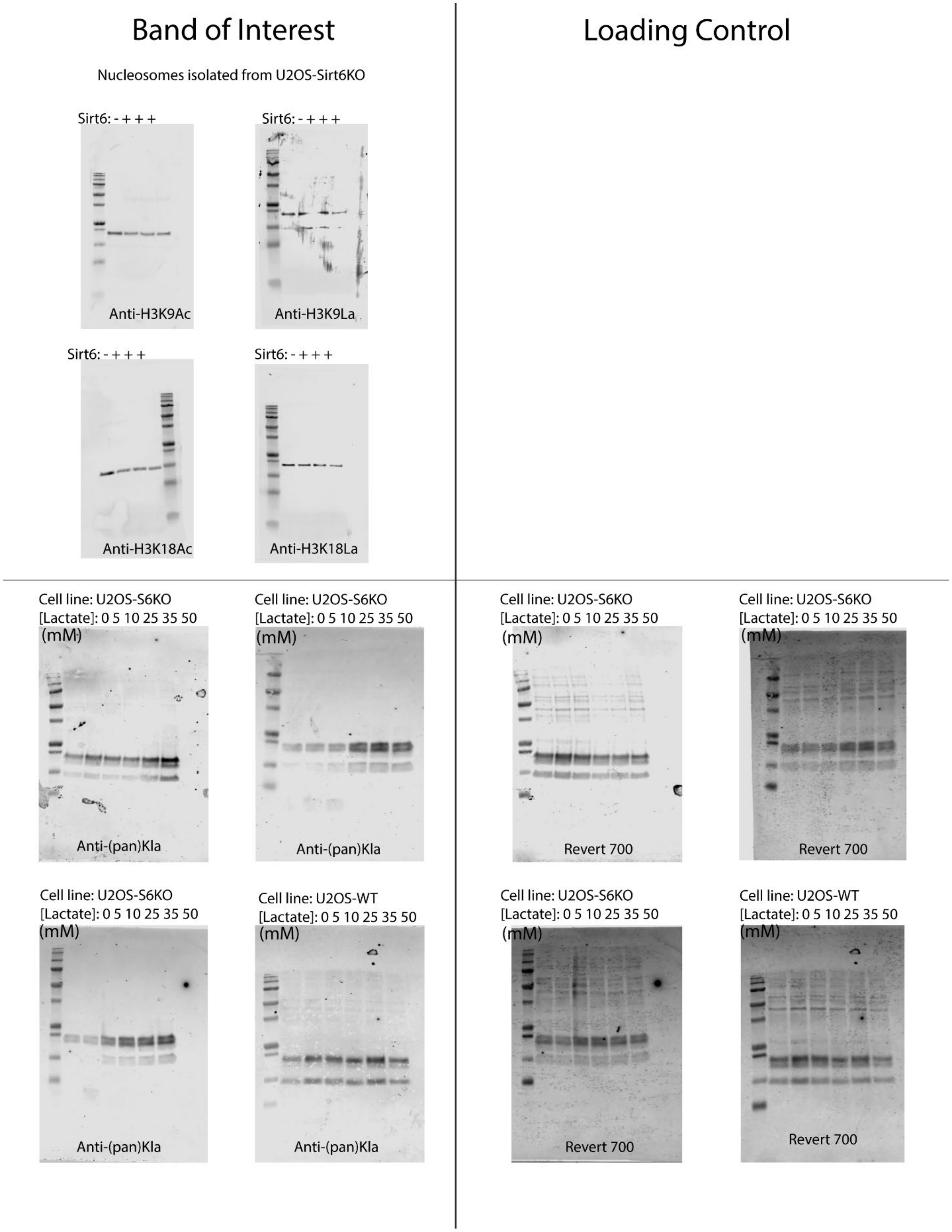
Band of Interest
Loading Control
Nucleosomes isolated from U2OS-Sirt6KO
Sirt6: - + + +
Anti-H3K9Ac
Sirt6: - + + +
Anti-H3K9La
Sirt6: - + + +
Anti-H3K18Ac
Sirt6: - + + +
Anti-H3K18La
Cell line: U2OS-S6KO
[Lactate]: 0 5 10 25 35 50
(mM)
Anti-(pan)Kla
Cell line: U2OS-S6KO
[Lactate]: 0 5 10 25 35 50
(mM)
Anti-(pan)Kla
Cell line: U2OS-S6KO
[Lactate]: 0 5 10 25 35 50
(mM)
Revert 700
Cell line: U2OS-S6KO
[Lactate]: 0 5 10 25 35 50
(mM)
Revert 700
Cell line: U2OS-S6KO
[Lactate]: 0 5 10 25 35 50
(mM)
Anti-(pan)Kla
Cell line: U2OS-WT
[Lactate]: 0 5 10 25 35 50
(mM)
Anti-(pan)Kla
Cell line: U2OS-S6KO
[Lactate]: 0 5 10 25 35 50
(mM)
Revert 700
Cell line: U2OS-WT
[Lactate]: 0 5 10 25 35 50
(mM)
Revert 700

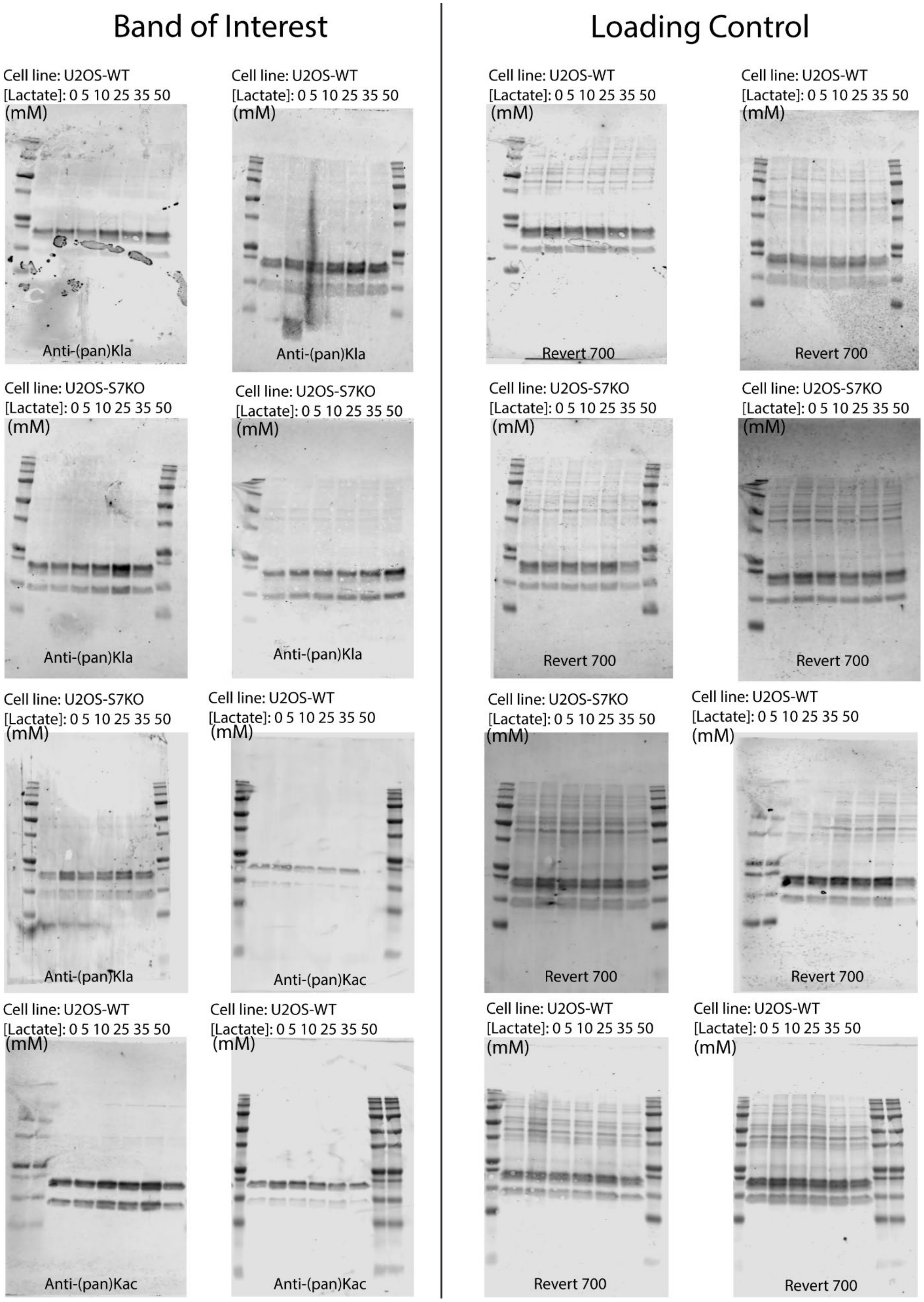
Band of Interest
Loading Control
Cell line: U2OS-WT
[Lactate]: 0 5 10 25 35 50
(mM)
Anti-(pan)Kla
Cell line: U2OS-WT
[Lactate]: 0 5 10 25 35 50
(mM)
Anti-(pan)Kla
Cell line: U2OS-WT
[Lactate]: 0 5 10 25 35 50
(mM)
Revert 700
Cell line: U2OS-WT
[Lactate]: 0 5 10 25 35 50
(mM)
Revert 700
Cell line: U2OS-S7KO
[Lactate]: 0 5 10 25 35 50
(mM)
Anti-(pan)Kla
Cell line: U2OS-S7KO
[Lactate]: 0 5 10 25 35 50
(mM)
Anti-(pan)Kla
Cell line: U2OS-S7KO
[Lactate]: 0 5 10 25 35 50
(mM)
Revert 700
Cell line: U2OS-S7KO
[Lactate]: 0 5 10 25 35 50
(mM)
Revert 700
Cell line: U2OS-S7KO
[Lactate]: 0 5 10 25 35 50
(mM)
Anti-(pan)Kla
Cell line: U2OS-WT
[Lactate]: 0 5 10 25 35 50
(mM)
Anti-(pan)Kac
Cell line: U2OS-S7KO
[Lactate]: 0 5 10 25 35 50
(mM)
Revert 700
Cell line: U2OS-WT
[Lactate]: 0 5 10 25 35 50
(mM)
Revert 700
Cell line: U2OS-WT
[Lactate]: 0 5 10 25 35 50
(mM)
Anti-(pan)Kac
Cell line: U2OS-WT
[Lactate]: 0 5 10 25 35 50
(mM)
Anti-(pan)Kac
Cell line: U2OS-WT
[Lactate]: 0 5 10 25 35 50
(mM)
Revert 700
Cell line: U2OS-WT
[Lactate]: 0 5 10 25 35 50
(mM)
Revert 700

Sirtuin 6 is a histone lysine delactylase (Supporting Information)

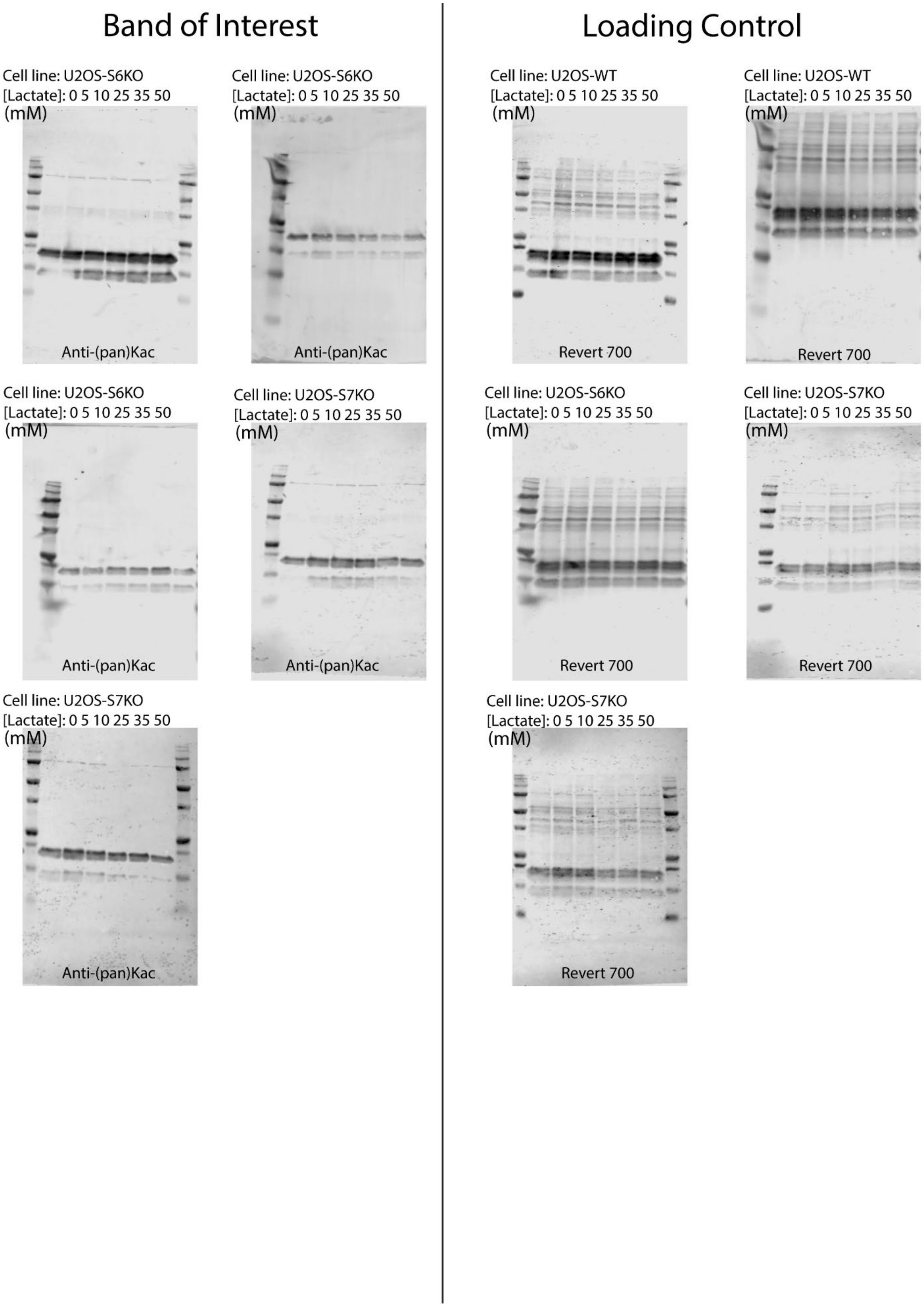

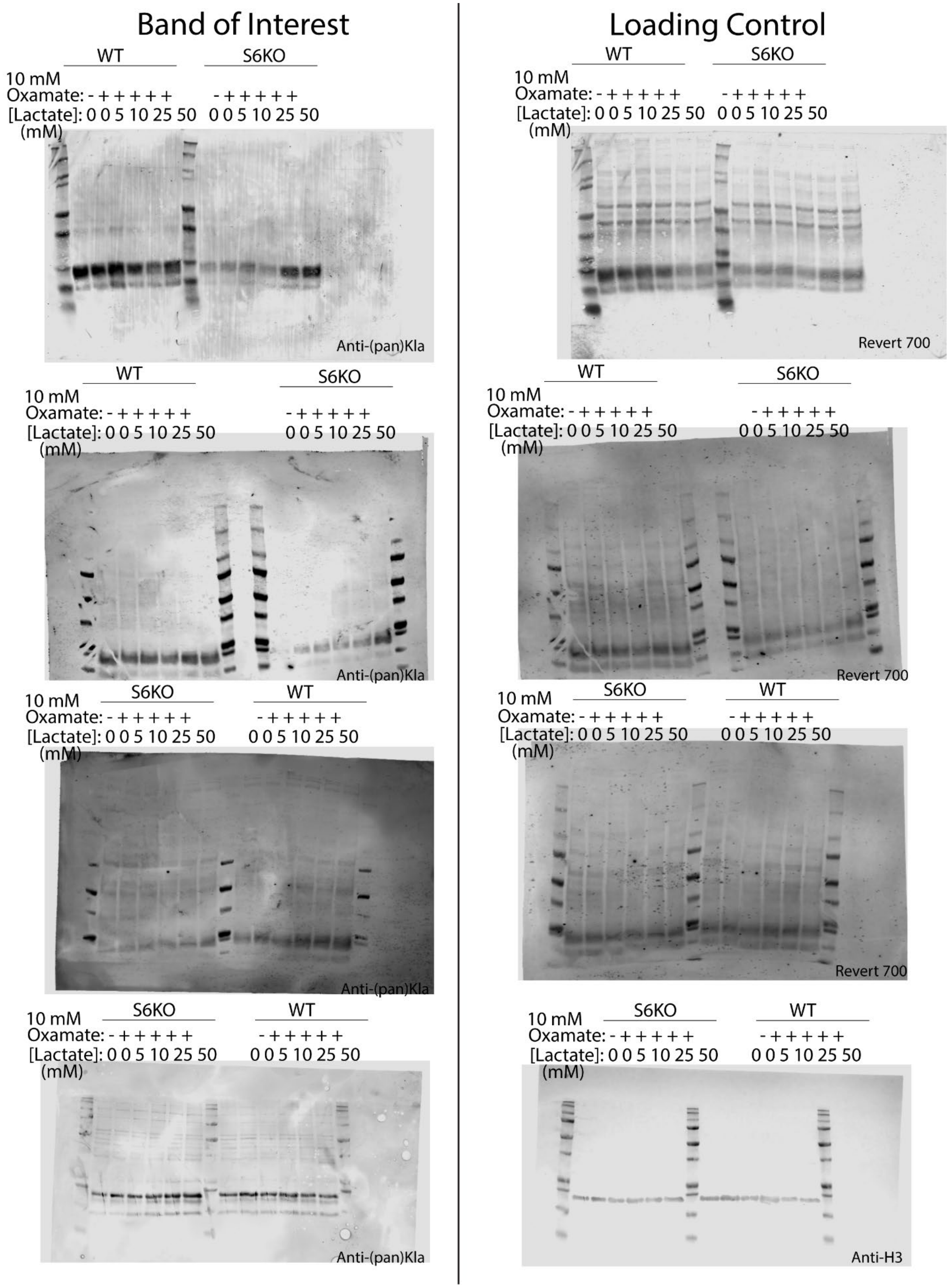
Band of Interest
Loading Control
WT
S6KO
10 mM
Oxamate: - + + + + +
[Lactate]: 0 0 5 10 25 50
(mM)
Anti-(pan)Kla
Revert 700
Anti-H3

Sirtuin 6 is a histone lysine delactylase (Supporting Information)

800 nM (Band of Interest)

Cell Line: U2OS WT
Rotenone (nM): 0 5 10 15 0 5 10 15 0 5 10 15

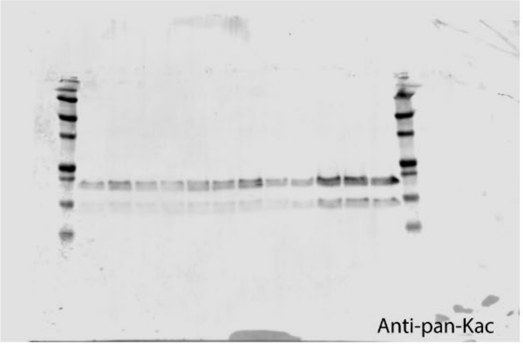

Cell Line: U2OS S6KO
Rotenone (nM): 0 5 10 15 0 5 10 15 0 5 10 15

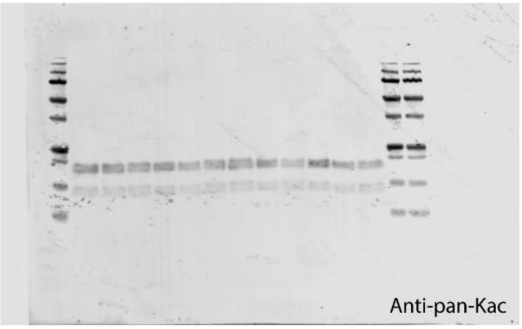

Cell Line: U2OS WT
Rotenone (nM): 0 5 10 15 0 5 10 15

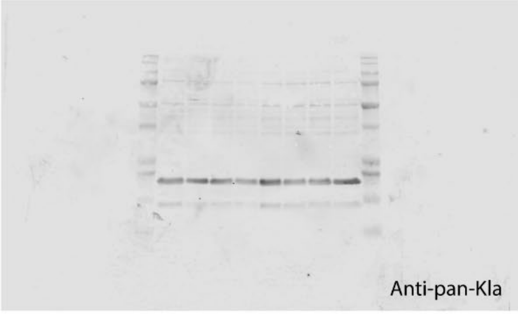

WT Sirt6-KO
Rotenone (nM): 0 5 10 15 0 5 10 15 0 5 10 15 0 5 10 15

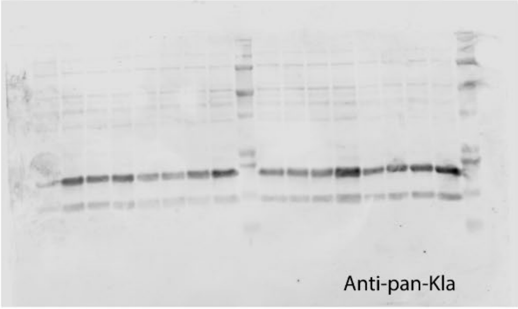

700 nM (Loading Control)

Cell Line: U2OS WT
Rotenone (nM): 0 5 10 15 0 5 10 15 0 5 10 15

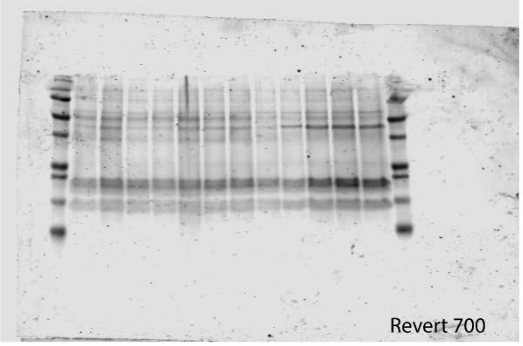

Cell Line: U2OS S6KO
Rotenone (nM): 0 5 10 15 0 5 10 15 0 5 10 15

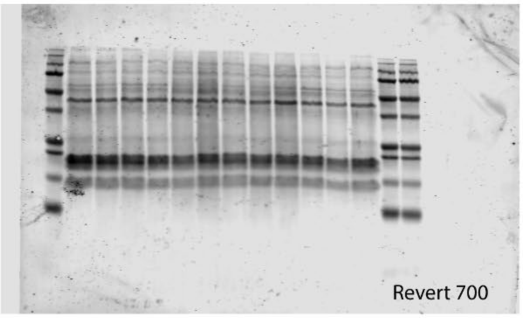

Cell Line: U2OS WT
Rotenone (nM): 0 5 10 15 0 5 10 15

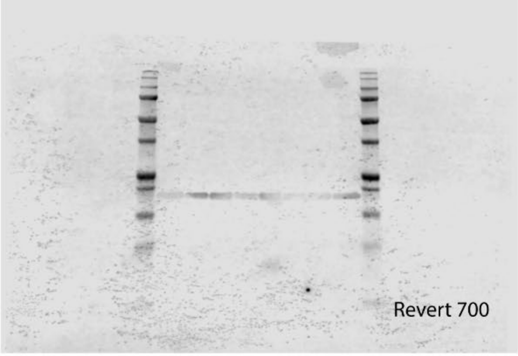

WT Sirt6-KO
Rotenone (nM): 0 5 10 15 0 5 10 15 0 5 10 15 0 5 10 15

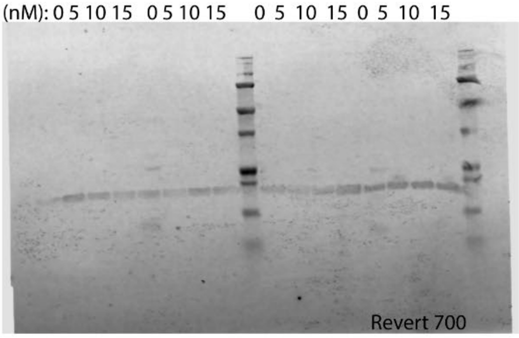

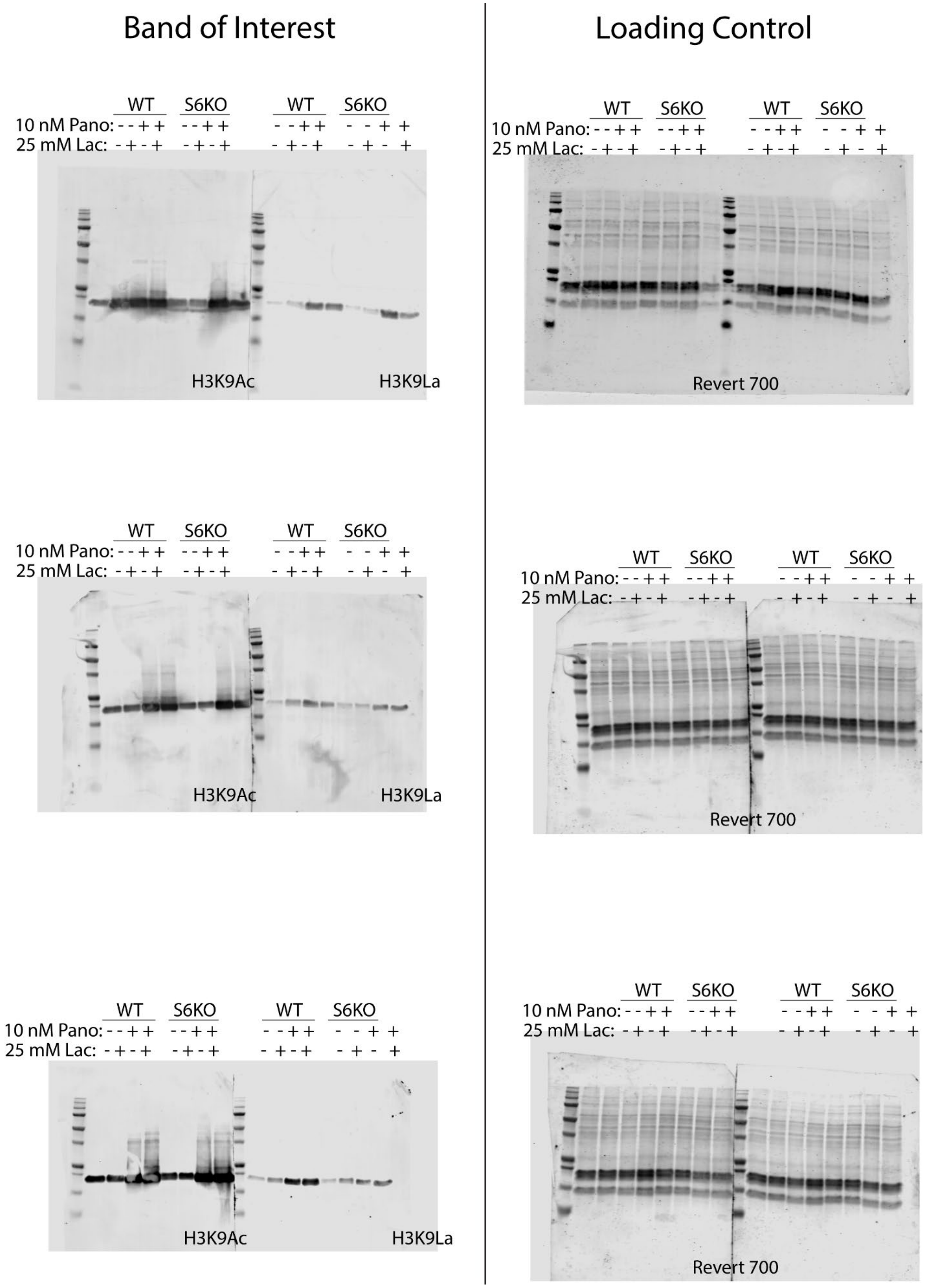
Band of Interest
Loading Control
WT
S6KO
10 nM Pano: - - + +
25 mM Lac: - + - +
H3K9Ac
H3K9La
Revert 700

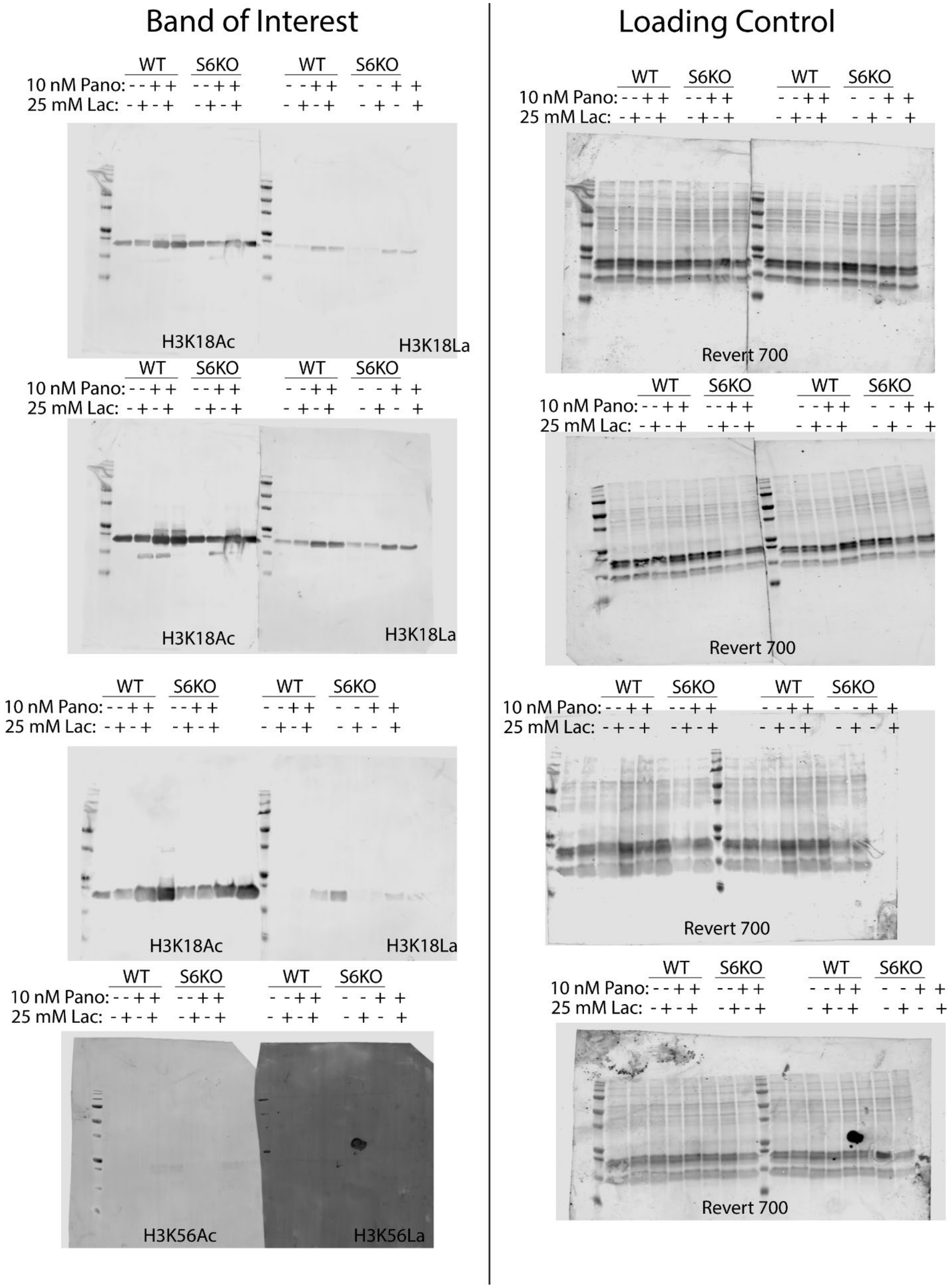
Band of Interest
Loading Control
WT S6KO WT S6KO
10 nM Pano: - - + + - - + + - - + + - - + +
25 mM Lac: - + - + - + - + - + - + - + - +
H3K18Ac
H3K18La
H3K56Ac
H3K56La
Revert 700

Sirtuin 6 is a histone lysine delactylase (Supporting Information)

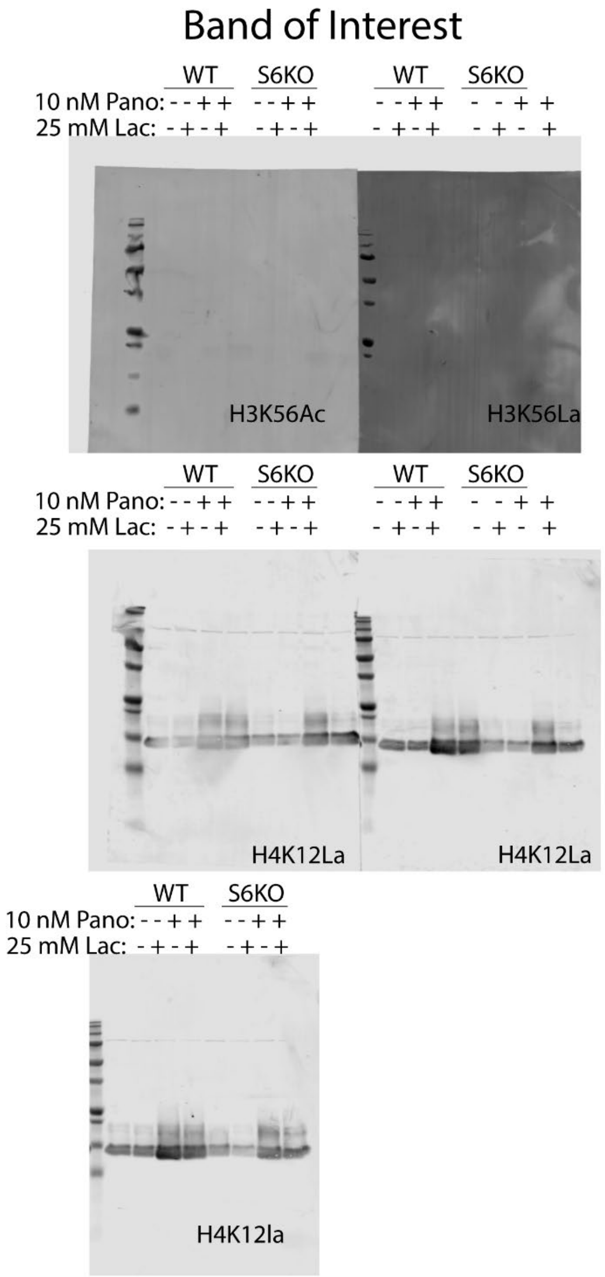

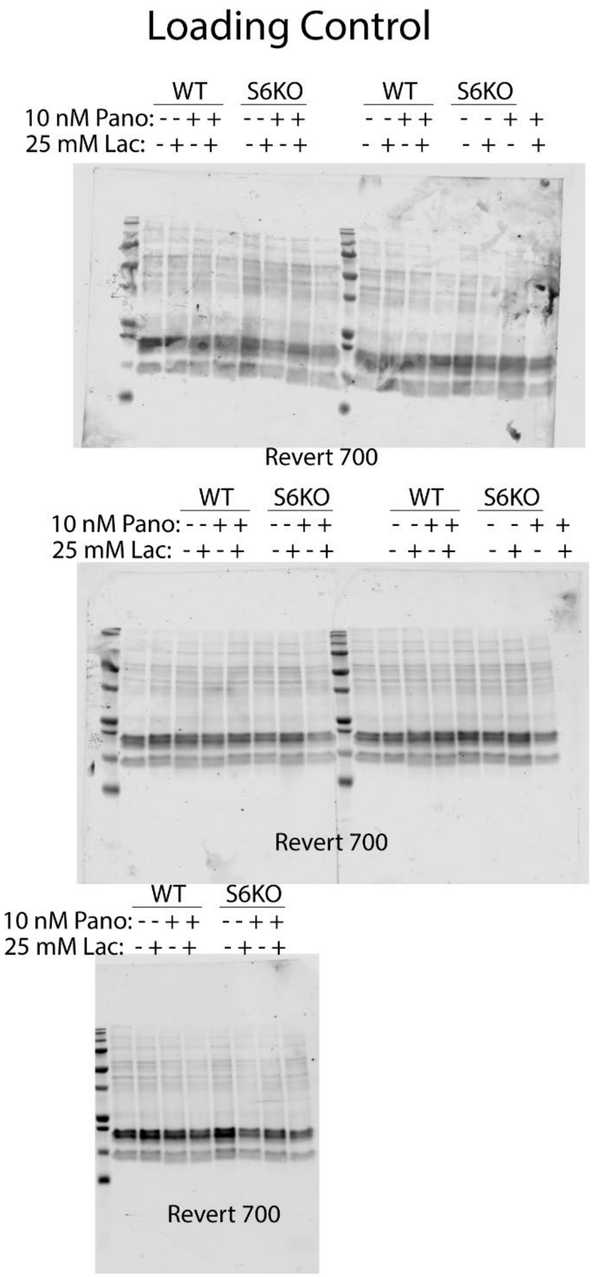

Sirtuin 6 is a histone lysine delactylase (Supporting Information)

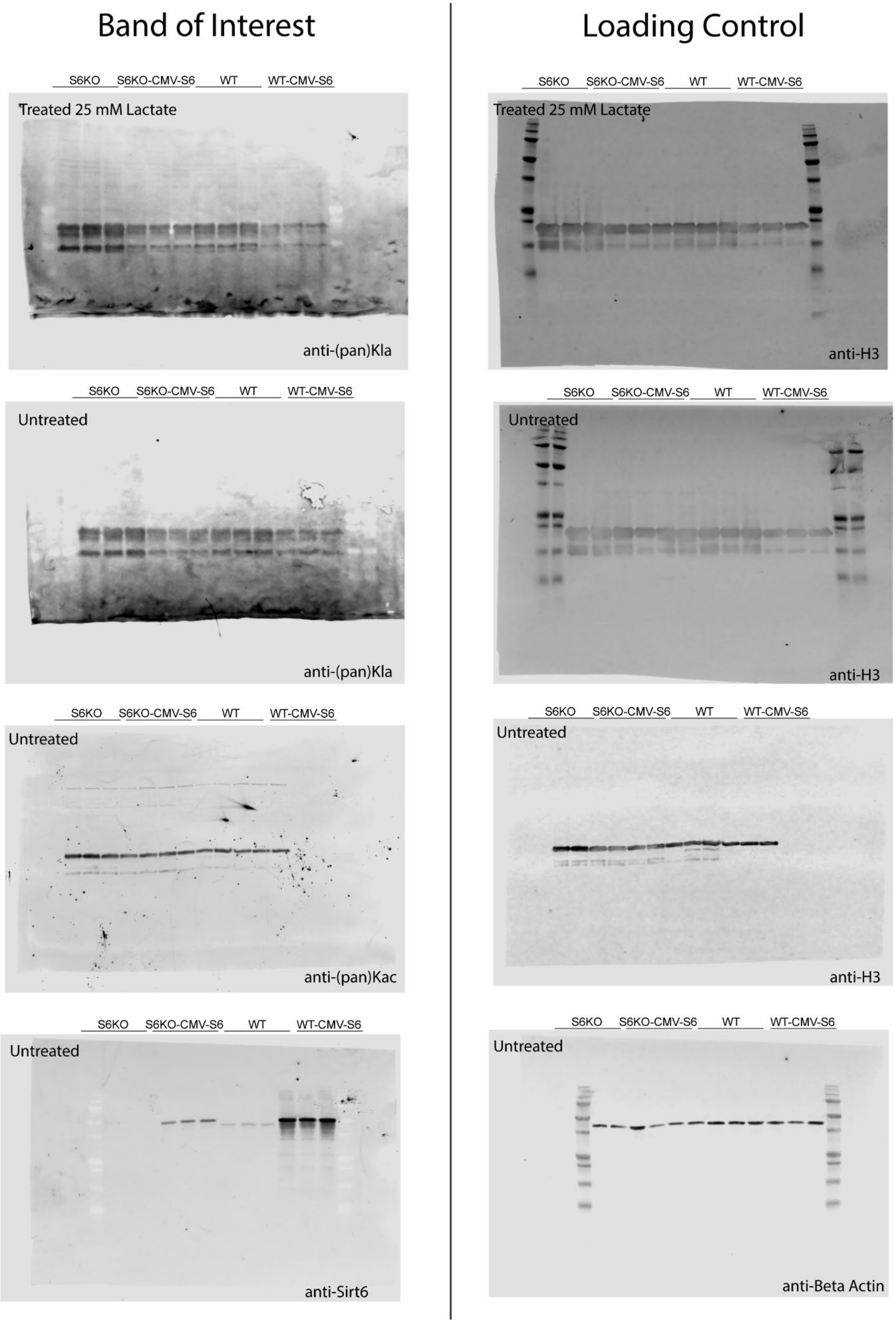

Sirtuin 6 is a histone lysine delactylase (Supporting Information)

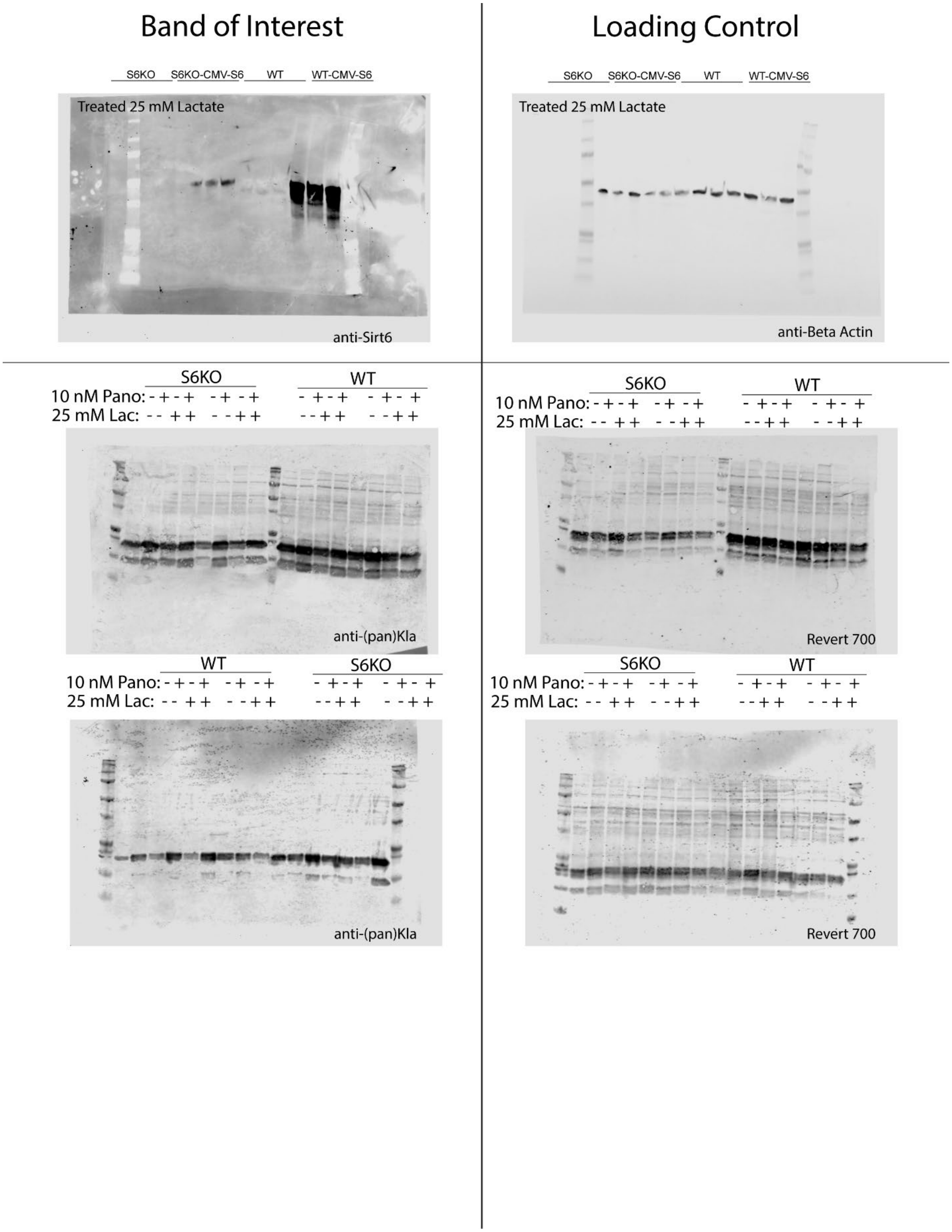

Sirtuin 6 is a histone lysine delactylase (Supporting Information)

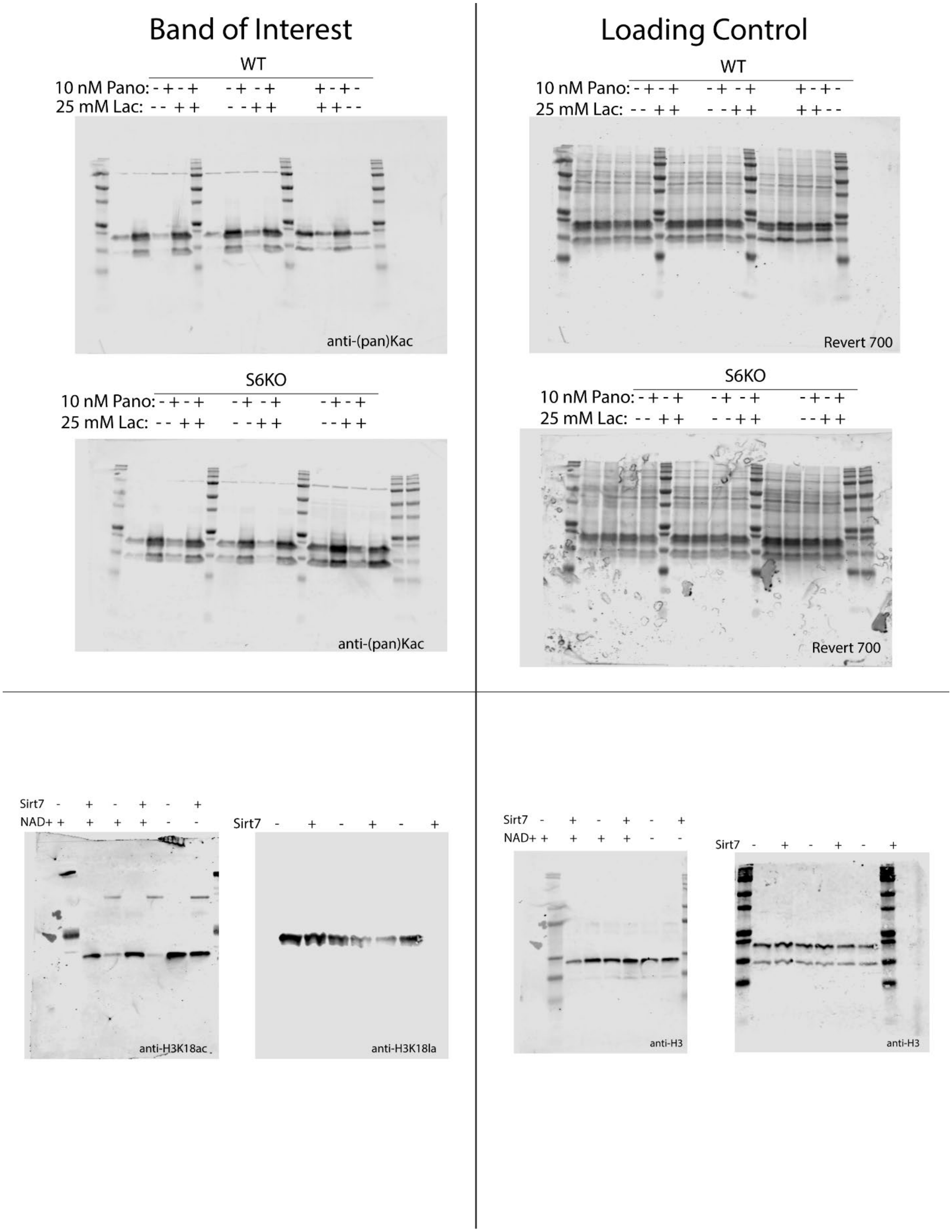

Sirtuin 6 is a histone lysine delactylase (Supporting Information)

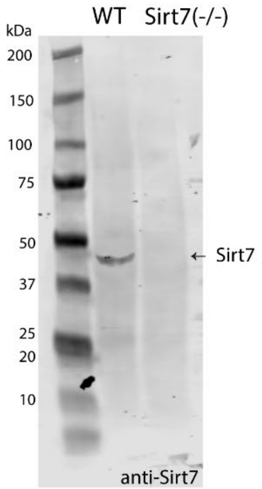

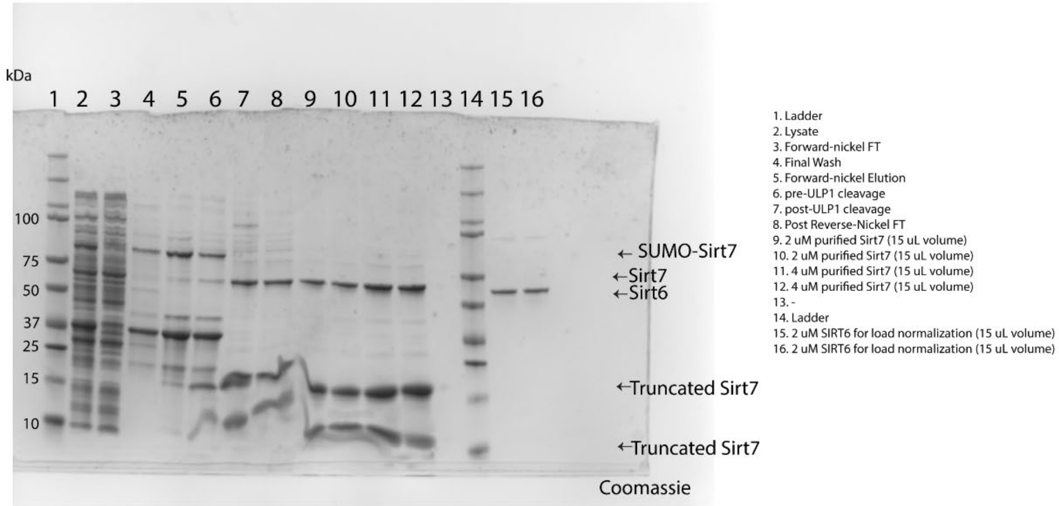

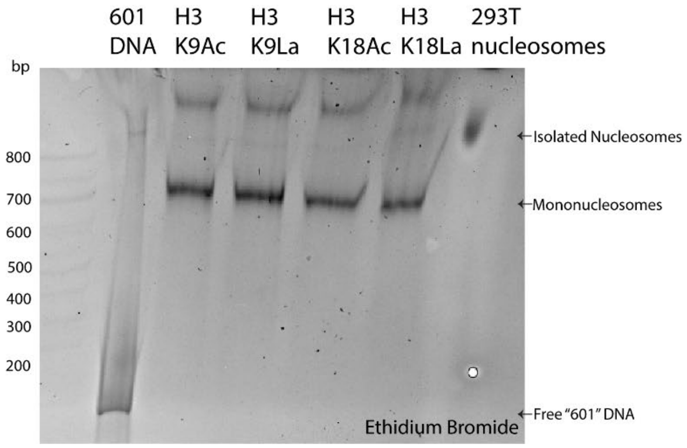

Supplement: Supporting Table and Figures [file mmc2.pdf]
